# Fast and selective labeling of N-terminal cysteines at neutral pH *via* thiazolidino boronate formation†

**DOI:** 10.1039/c6sc00172f

**Published:** 2016-04-04

**Authors:** Anupam Bandyopadhyay, Samantha Cambray, Jianmin Gao

**Affiliations:** a Department of Chemistry, Merkert Chemistry Center, Boston College 2609 Beacon Street Chestnut Hill MA 02467 USA jianmin.gao@bc.edu

## Abstract

Facile labeling of proteins of interest is highly desirable in proteomic research as well as in the development of protein therapeutics. Herein we report a novel method that allows for fast and selective labeling of proteins with an N-terminal cysteine. Although N-terminal cysteines are well known to conjugate with aldehydes to give thiazolidines, the reaction requires acidic conditions and suffers from slow kinetics. We show that benzaldehyde with an *ortho*-boronic acid substituent readily reacts with N-terminal cysteines at neutral pH, giving rate constants on the order of 10^3^ M^−1^ s^−1^. The product features a thiazolidino boronate (TzB) structure and exhibits improved stability due to formation of the B–N dative bond. While stable at neutral pH, the TzB complex dissociates upon mild acidification. These characteristics make the TzB conjugation chemistry potentially useful for the development of drug–protein conjugates that release the small molecule drug in acidic endosomes.

## Abstract 2

Facile labeling of proteins of interest is highly desirable in proteomic research as well as in the development of protein therapeutics.

## Graphical abstract

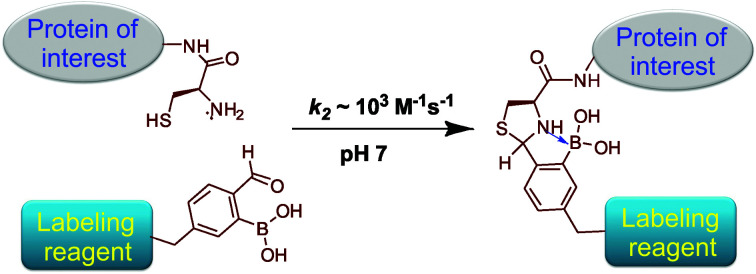

## Introduction

Methods that allow facile labeling of proteins of interest have been heavily sought-after towards the goal of defining the functions of individual proteins in cells.^1^ On the other hand, the development of protein-based therapeutics requires both protein modification and labeling, ideally in a site-specific manner.^2^ Much progress has been made in the field of bioorthogonal chemistry,^3^ which allows site-specific labeling of proteins that incorporate unnatural amino acids as handles. However, it would be advantageous if natural proteinogenic amino acids could be targeted for modification. Toward this end, several enzyme-mediated labeling strategies have been reported,^4–6^ in which designed enzymes recognize specific peptide sequences for conjugation. These approaches are less ideal due to their need for exogenous enzymes. It remains a challenge to label proteins of interest with site specificity and in native biological settings, save two examples that take advantage of a tetra-cysteine motif^7^ and a cysteine sitting in a π-clamp,^8^ respectively.

**Fig. 1:**
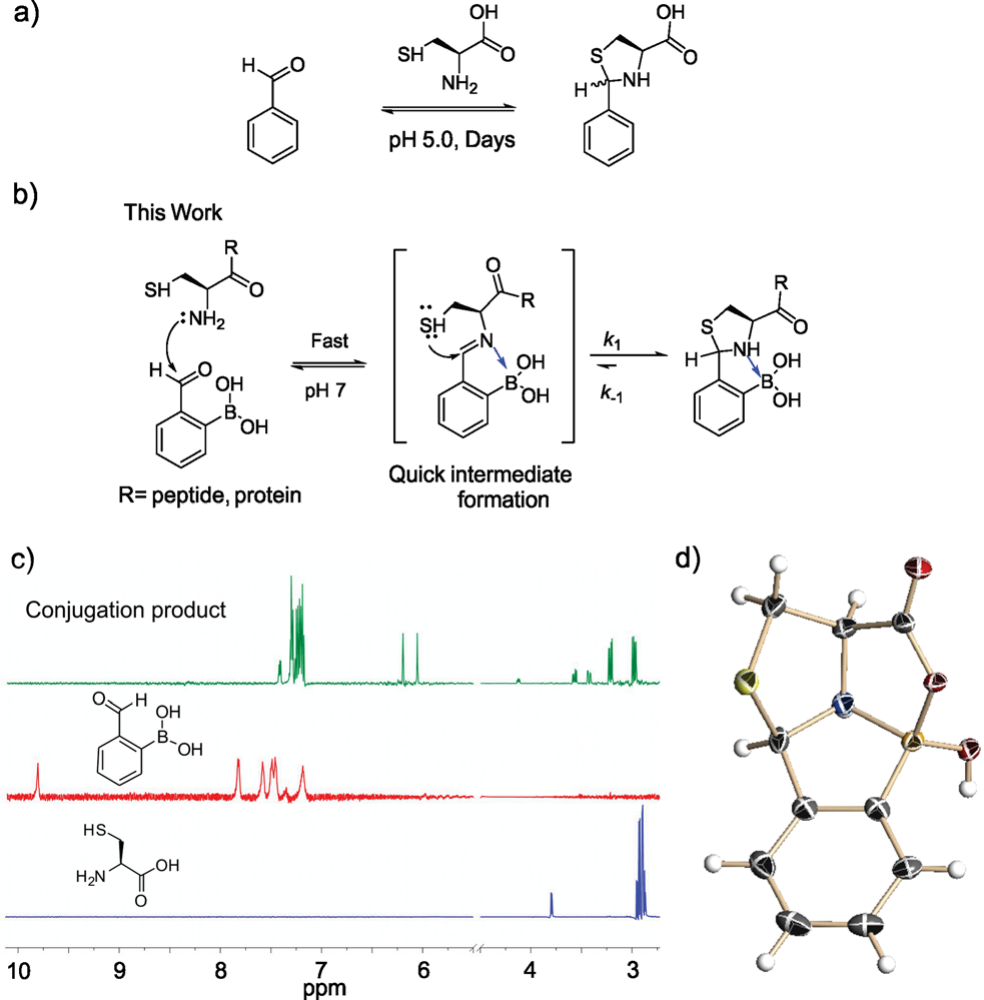
Thiazolidino boronate (TzB) formation of cysteine. (a) Illustration of cysteine modification *via* thiazolidine formation. (b) Illustration of the boronic acid-accelerated cysteine modification to give a thiazolidino boronate (TzB) complex. (c) ^1^H-NMR data showcasing the fast and clean conjugation between 2-FPBA and l-cysteine. (d) Crystal structure of the TzB complex formed between 2-FPBA and l-cysteine.

Although commonly targeted for protein labeling, a cysteine residue cannot afford protein specificity or site specificity in complex biological mixtures because many endogenous proteins would present multiple cysteine residues. However, when positioned at the N-terminus of a protein, a cysteine residue may be selectively targeted because it presents a distinctive 1,2-aminothiol functionality. It is well known that an N-terminal cysteine can selectively react with aldehydes to form thiazolidines with no interference from other nucleophilic residues such as serines, lysines, and even internal cysteines.^9–11^ However, this reaction requires acidic conditions (pH 4–5) and suffers from slow kinetics: it is typically performed with high concentrations of reactants and long incubation times (∼2 days), even at pH 5 (Fig. 1a).^12,13^

In this contribution, we report a protocol for rapid and selective modification of N-terminal cysteines using benzaldehyde carrying an *ortho*-boronic acid substituent. The boronic acid promotes facile thiazolidine formation at neutral pH, which gives rate constants greater than 10^3^ M^−1^ s^−1^ and affords one of the fastest bioorthogonal reactions for protein labeling (Fig. 1b).

## Results and discussions

Recently, we^14–16^ and others^17–21^ have demonstrated the thermodynamic and kinetic benefit of an *ortho*-boronic acid moiety in the formation of imines, as well as oximes and hydrazones. As thiazolidine formation potentially goes through an imine intermediate, we hypothesized that a boronic acid moiety installed at the *ortho* position of benzaldehyde would be able to activate the imine to facilitate thiazolidine formation (Fig. 1b). To test our hypothesis, an equimolar mixture of 2-formyl phenylboronic acid (2-FPBA, 1 mM) and l-cysteine was prepared in a pH 7 buffer and the reaction was analysed by NMR spectroscopy and mass spectrometry. In ^1^H-NMR characterization, a fast and clean conversion was observed as the 2-FPBA resonances completely disappeared in less than 10 min (Fig. 1c). In contrast, the unsubstituted benzaldehyde showed no reaction with cysteine even after 3 h (Fig. S1†).

The conjugation product of 2-FPBA and cysteine exhibits two sets of peaks in ^1^H-NMR at pH 7. For example, two singlets are observed around 6 ppm, where the benzylic proton of the thiazolidine product is expected (Fig. 1c). The ^1^H-NMR data indicate the existence of two species in the conjugation product. However, X-ray crystallography data revealed a single diastereomer exhibiting a polycyclic structure (Fig. 1d), in which formation of a B–N dative bond (1.66 Å) affords a thiazolidino boronate (TzB) complex. Lending further support to the TzB complex formation, the ^11^B-NMR spectrum displays peaks around 10 ppm, which is expected for the partial anionic boron in boronate structures (Fig. S2†).^15^ Interestingly, the crystal structure shows that a mixed anhydride is formed between the cysteine –COOH and the boronic acid. It is thought that the B–N and B–O bond formation preorganizes the conjugate structure and results in the thiol attack of the imine from the top face to give the single diastereomer observed. To further elucidate the nature of the two species observed in NMR, we performed a pH titration experiment using both ^1^H and ^11^B-NMR. The results show that the two species observed at pH 7 readily interconvert upon pH variation to give predominantly one species at pH 5.5 and the other at pH 7.8 (Fig. S2†). The pH dependent behaviour indicates that the second species observed in NMR most likely result from hydrolysis of the mixed anhydride under slightly basic conditions. Indeed, mass-spec analysis revealed the molecular ions that correspond to the hydrolysed product, as well as the mixed anhydride (Fig. S3†).

**Fig. 2:**
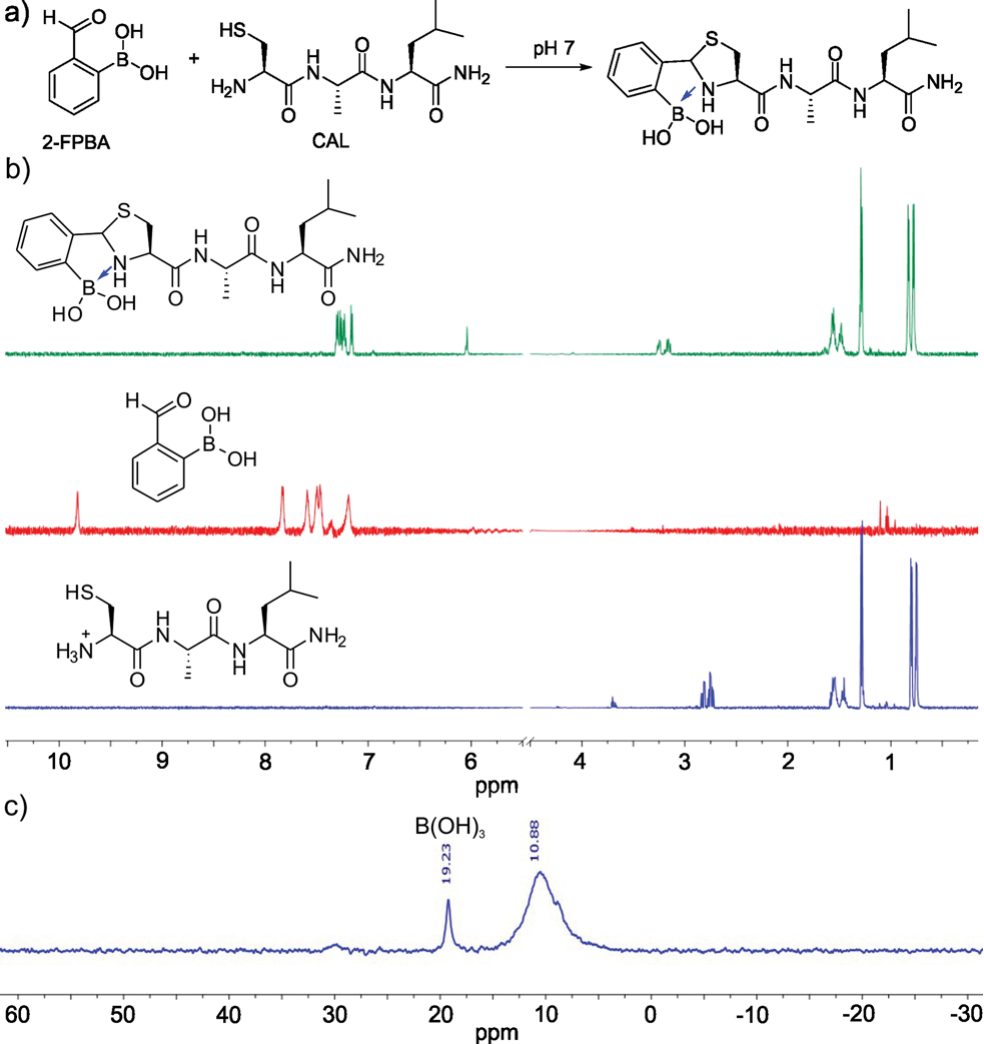
Conjugation of 2-FPBA to peptides with N-terminal cysteines. (a) The reaction scheme pf 2-FPBA and a tri-peptide CAL conjugation. (b) ^1^H-NMR data demonstrating fast and clean conjugation between 2-FPBA and CAL. (c) ^11^B-NMR illustrating the formation of a TzB complex.

Encouraged by the facile conjugation between 2-FPBA and cysteine, we explored the potential of using 2-FPBA to label peptides and proteins with N-terminal cysteines. Toward this end, we first examined a short peptide CAL (Fig. 2a) as a model system. The peptide was mixed with 2-FPBA at a 1 : 1 ratio in a pH 7 buffer (1 mM final concentration). Similar to what we observed for free cysteine, the peptide CAL readily conjugated with 2-FPBA according to ^1^H-NMR, which showed complete disappearance of the aldehyde peak in less than 10 min. A new peak appeared at ∼6 ppm, which is characteristic of thiazolidine formation (Fig. 2b). Interestingly, for the 2-FPBA–CAL conjugate, only a single peak was observed at 6 ppm, which differs from that of free cysteine (Fig. 1c). This difference is presumably due to the fact that the N-terminal cysteine in CAL can no longer form a mixed anhydride with the boronic acid. Nevertheless, the single peak at 6 ppm indicates only one diastereomer is obtained the 2-FPBA–CAL conjugation. This result suggests that the B–N dative bond formation dictates the stereochemistry of thiazolidine formation. ^11^B-NMR of the 2-FPBA–CAL conjugate shows a major peak around 10 ppm (Fig. 2c), indicating formation of a TzB complex similar to what we observed for free cysteine. Mass-spec analysis supports formation of the TzB complex between 2-FPBA and CAL as well (Fig. S4†).

**Fig. 3:**
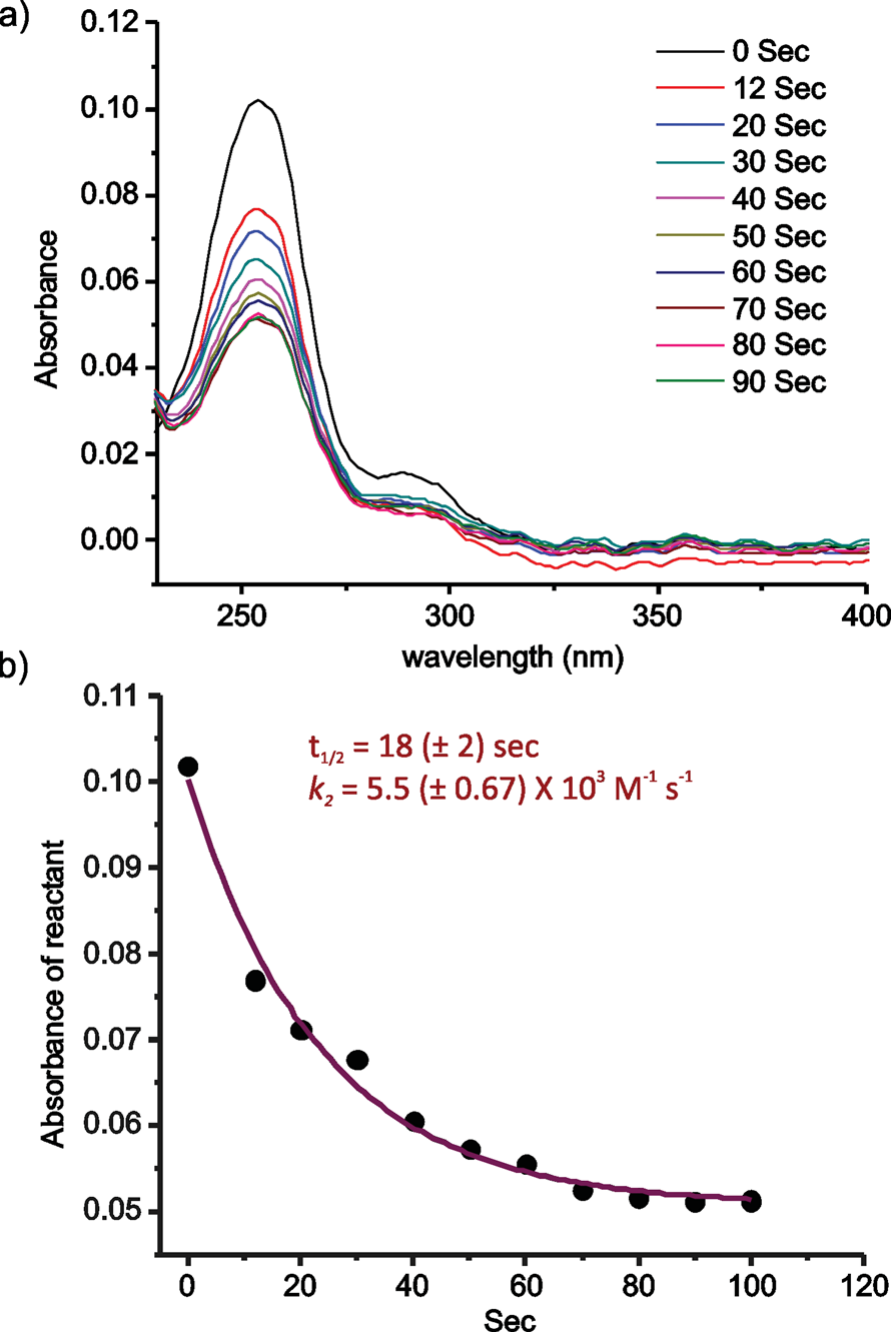
Kinetics of 2-FPBA–CAL conjugation. (a) UV-vis absorption changes over time after mixing of 2-FPBA and CAL (pH 7, 10 μM each). (b) Example profile of the 2-FPBA–CAL conjugation kinetics. Data fitting yields the *t*_1/2_ values and the rate constants. Three separate measurements were carried out, from which the means of *t*_1/2_ and *k*_2_ were obtained together with their error values (shown in parentheses).

The kinetics of the 2-FPBA–CAL conjugation was quantitatively assessed *via* a UV-vis experiment, which allows the reaction to be monitored at low concentrations (Fig. 3). 2-FPBA exhibits an absorption maximum at 254 nm, which decreases significantly upon conversion of the aldehyde to a thiazolidine. For the kinetics measurement, 2-FPBA and CAL were mixed at 10 μM each. At this concentration, essentially complete conjugation can be achieved according to a titration experiment (Fig. S5†). The reaction was monitored by recording the absorption decrease over time (Fig. 3a). The results show that the conjugation completed to 50% within only 18 seconds, which is remarkably fast considering the low concentrations of the reactants used. Fitting the data according to a second order kinetics mechanism gives a rate constant (*k*_2_) of 5.5 × 10^3^ M^−1^ s^−1^, which is comparable to some of the fastest bioorthogonal reactions documented in literature (Fig. 3b).^15,18,22–24^

**Fig. 4:**
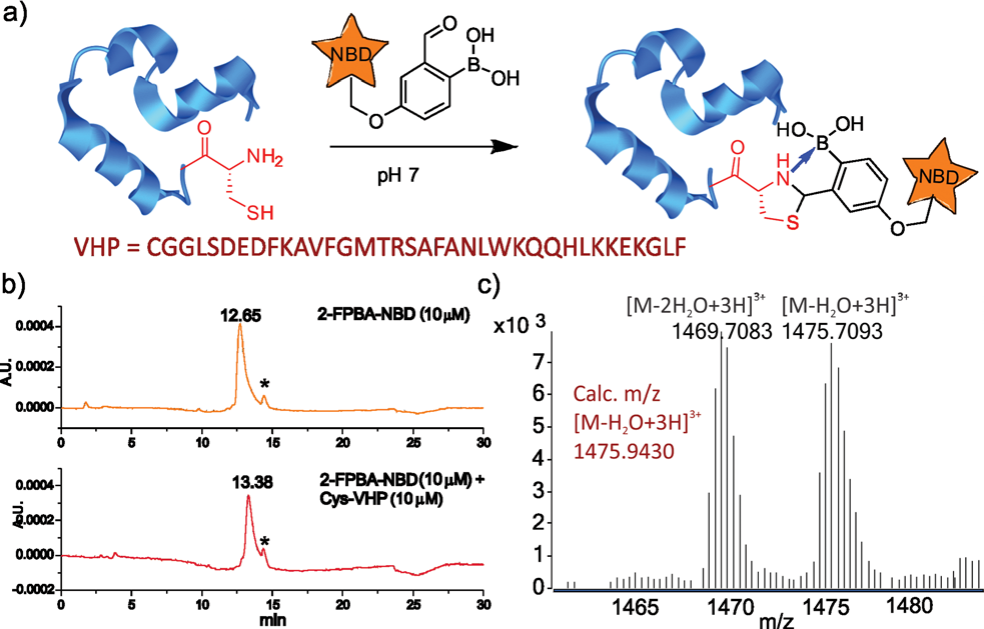
Facile labeling of a model protein *via* TzB complex formation. (a) Illustration of N-terminal labeling of Cys-VHP with 2-FPBA–NBD. (b) LC traces monitored at 460 nm and (c) mass spec results showing complete conjugation of 2-FPBA–NBD to Cys-VHP. The minor peaks marked with * in (b) originate from minor oxidation of 2-FPBA–NBD, which converts the boronic acid to a hydroxyl.

To further demonstrate the utility of the TzB conjugation chemistry for protein labeling, we synthesized a fluorophore-labelled derivative of 2-FPBA (2-FPBA–NBD, see ESI† for details), as well as a small model protein villin headpiece subdomain bearing a cysteine at its N-terminus (Cys-VHP, Fig. 4a). The labeling of Cys-VHP by 2-FPBA–NBD was examined by mixing them at 10 μM concentration in a pH 7 buffer. After 30 min incubation, the reaction mixture was analysed *via* LC-MS. The result shows essentially complete conversion of 2-FPBA–NDB to its VHP conjugate (Fig. 4b), the identity of which was confirmed *via* mass-spec analysis (Fig. 4c and S6†).

**Fig. 5:**
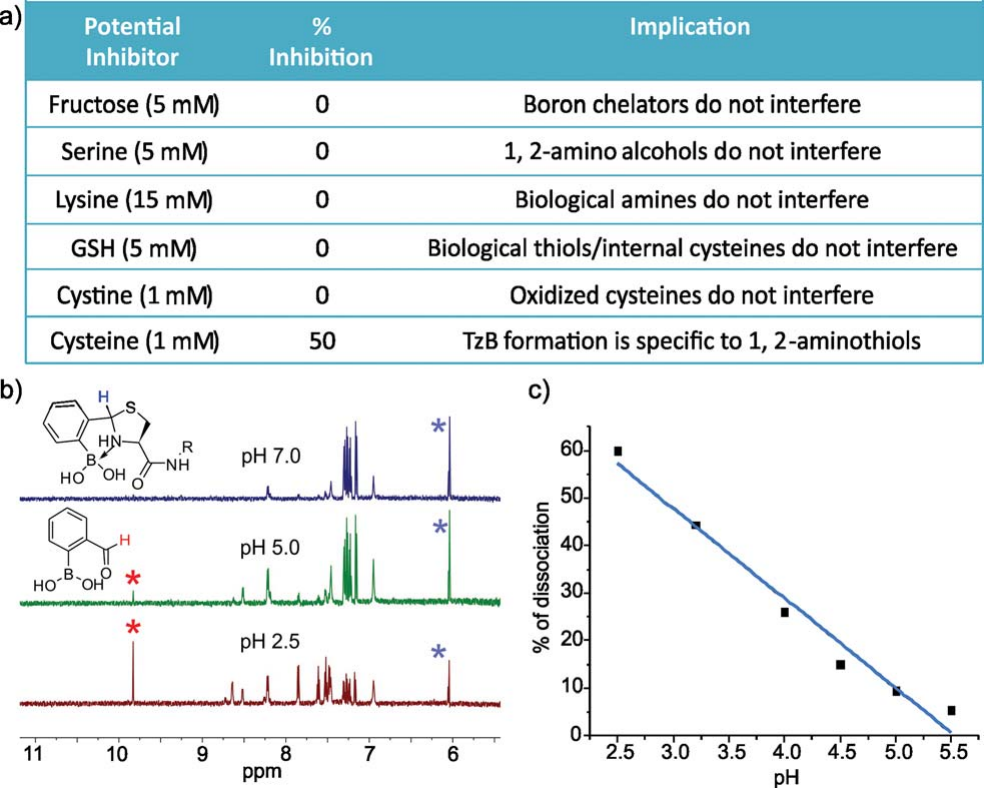
Stability and pH-triggered reversibility of the TzB conjugate between 2-FPBA and CAL. (a) List of biologically relevant small molecules and their effect (or lack of thereof) on TzB complex formation. (b) A collection of ^1^H-NMR spectra showing the acid-triggered dissociation of the 2-FPBA–CAL conjugate. (c) Extent of dissociation of the 2-FPBA–CAL complex under various pH conditions.

To explore the application of TzB chemistry in complex biological systems, we assessed the stability of 2-FPBA-labeled peptides during purification, in storage, and in the presence of various abundant biomolecules. First, our results show that the 2-FPBA labelled peptides (CAL and Cys-VHP) can be easily purified through HPLC by using acid-free eluents (Fig. S7 and 8†). The 2-FPBA–CAL conjugate was chosen for further stability studies because its simple structure makes it amenable to ^1^H-NMR analysis. Specifically, the 2-FPBA–CAL conjugate was dissolved in a neutral buffer and its integrity was periodically examined by ^1^H-NMR. The results show that the conjugate remained intact, even after five days (Fig. S7†). In contrast, the CAL conjugate with salicylaldehyde gave ∼25% dissociation after 10 hours (Fig. S9†). The improved stability of the 2-FPBA–CAL conjugate presumably originates from the B–N dative bond in the TzB complex. We further examined the conjugation efficacy of 2-FPBA and CAL in presence of various biomolecules. Remarkably, ^1^H-NMR studies found that TzB conjugation was not affected by a range of molecules that are commonly seen in biology (Fig. 5a and S10†), including fructose (5 mM), serine (5 mM), lysine (15 mM), glutathione (GSH, 5 mM) and cystine (1 mM). These results nicely showcase the high specificity of the TzB conjugation chemistry towards 1,2-aminothiols. Lending further support to this statement, 2-FPBA elicited no detectable conjugation with a preorganized Cys–Lys pair in a helical peptide (Fig. S11†). Not surprisingly, adding free cysteine at equimolar concentration (1 mM) resulted in ∼50% conversion of the 2-FPBA–CAL conjugate to the 2-FPBA–cysteine conjugate, and the cysteine–CAL exchange completed over the course of two hours (Fig. S12†). These data suggest 2-FPBA labelled proteins may slowly exchange with free cysteine. However, we note that free cysteine only exists at low μM concentrations in blood serum,^25^ while cysteine as the major species does not compromise the integrity of the TzB complex.

Various protein modifications that can be reversed in a well-controlled manner have been adopted by nature to regulate protein function; a prominent example is protein phosphorylation.^26^ Reversible protein modification has also proven beneficial to the development of protein therapeutics such as antibody–drug conjugates (ADCs).^12^ Considering the endocytotic mechanism of cell entry for protein therapeutics,^27^ a pH-triggered dissociation of the small molecule drug from the protein carrier would be ideal as endosomes present a mildly acidic environment. With these considerations, we took the 2-FPBA–CAL conjugate as a model TzB complex and assessed its dissociation potential under acidic conditions (Fig. 5b and c). The integrity of the 2-FPBA–CAL conjugate was quantified *via*^1^H-NMR under a range of pH conditions. The results show that the TzB complex of 2-FPBA and CAL remains intact at pHs above 6 (Fig. S13†). Mild acidification to pH 5 and 4 causes about 10% and 26% dissociation respectively. The dissociation appears to proceed rapidly as the ^1^H-NMR data suggest the reaction mixture reaches equilibrium as soon as the pH is tuned and the spectrum is taken (∼10 min, Fig. S14†). This fast and pH-triggered reversibility of the TzB complex formation makes it potentially useful for conjugating small molecule drugs to antibodies and other protein therapeutics, for which a number of strategies have been reported for the preparation of recombinant proteins with N-terminal cysteines.^13,28,29^

## Conclusions

This contribution describes a fast and selective conjugation chemistry of N-terminal cysteines. By installing an *ortho*-boronic acid functionality, the conjugation of benzaldehyde and an N-terminal cysteine is greatly accelerated through formation of an iminoboronate intermediate, in which the boronic acid activates the imine for thiazolidine formation. The conjugation chemistry exhibits little interference by abundant biomolecules (fructose, serine, lysine, glutathione, cystine) and gives second order rate constants on the order of 10^3^ M^−1^ s^−1^ at neutral pH. This is much more advantageous in comparison to the unsubstituted benzaldehyde, which shows sluggish reactivity with N-terminal cysteines, even under acidic conditions.^12^ Furthermore, the final product was found to exhibit superior stability due to boron coordination by the thiazolidine ring to give a thiazolidino boronate (TzB) complex. While the TzB complex is stable at neutral physiological conditions, it rapidly dissociates upon mild acidification to the pH seen in endosomes. Related to this work, an elegant conjugation chemistry of N-terminal cysteines has been reported in recent literature that takes advantage of the unique reactivity of cyanobenzothiazole towards 1,2-aminothiols.^30,31^ In comparison, the TzB complex formation described here enjoys faster kinetics and pH-triggered reversibility. These features make the TzB chemistry potentially useful for the development of antibody–drug conjugates that can release drugs in endosomes.

## Supplementary Material

SC-007-C6SC00172F-s001

SC-007-C6SC00172F-s002
