# Supplementary material for: Fast and selective labeling of N-terminal cysteines at neutral pH via thiazolidino boronate formation
Source: Chem Sci. 2016 Apr 4;7(7):4589–93. doi: 10.1039/c6sc00172f (PMC5201210; doi:10.1039/c6sc00172f)
Supplement: SC-007-C6SC00172F-s001 [file SC-007-C6SC00172F-s001.pdf]

Electronic Supplementary Material (ESI) for Chemical Science.
This journal is © The Royal Society of Chemistry 2016

Supplementary Material

Fast and selective labeling of N-terminal cysteines at neutral pH via thiazolidino boronate formation

Anupam Bandyopadhyay, Samantha Cambray and Jianmin Gao*

Department of Chemistry, Merkert Chemistry Center, Boston College, 2609 Beacon Street, Chestnut Hill, MA 02467

*To whom correspondence should be addressed. Email: jianmin.gao@bc.edu

Table of contents **Pages**

General methods	S2
Conjugation of benzaldehyde and L-cysteine	S3
Conjugation of 2-FPBA and L-cysteine	S3
NMR studies of the TzB complex formation between 2-FPBA and L-cysteine	S4
Conjugation of 2-FPBA and CAL	S5
Kinetics study of the TzB complex formation between 2-FPBA and CAL	S7
Protein labeling experiments	S8
Purification and storage method of TzB complex	S9
Comparing the stability of the 2-FPBA-CAL TzB complex and the salicylaldehyde-CAL thiazolidine conjugate	S12
Small molecule inhibition experiments for TzB complex formation	S13
Testing the reactivity of 2-FPBA toward a spatially preorganized Cys-Lys pair in α-helix	S15
L-cysteine exchange with a TzB conjugate	S16
Dissociation of a TzB conjugate in acidic pH	S18
Crystallographic information for 2-FPBA-cysteine thiazolidino boronate conjugate	S20
Synthesis of thiazolidine conjugate between salicylaldehyde and the CAL peptide	S23
Synthesis of Peptides and characterization	S24
Synthesis of NBD labelled 2-FPBA (2-FPBA-NBD)	S25
References	S28
NMR data	S29

I. General methods

2-formylphenylboronic acid (2-FPBA), 2-Bromo-5-hydroxybenzaldehyde, L-cysteine, L-serine, L-lysine, L-cystine, glutathione, benzaldehyde, salicylaldehyde and fructose were purchased from Fischer. Rink Amide MBHA resin and HBTU were purchased from Novabiochem (San Diego, CA). Fmoc-protected amino acids were purchased from Advanced Chemtech (Louisville, KY) or Chem-Impex Int'l Inc (Wood Dale, IL). A Nanodrop UV-vis spectrometer was used to measure the concentration of the NBD labelled compound. NMR data were collected on VNMRS 500 & 600 MHz NMR spectrometers. Mass-spec data were generated by an Agilent 6230 LC TOF mass spectrometer. Peptide synthesis was carried out on a Tribute peptide synthesizer from Protein Technologies. All the NMR studies, inhibition experiments and exchange experiments related to cysteine conjugation were performed in phosphate buffer pH = 7.0 (10 mM).

The gradients used for analytical HPLC and LC-MS analysis:
Method I for LC-MS (Agilent 6230 LC TOF): Agilent Extend C18 (1.8 µm, 2.1 × 50 mm) analytical column using water-acetonitrile mobile phase with a flow rate of 0.2 mL/min. Gradient used: isocratic 5% CH_3CN for 3 min (0-3 min), then gradient from 5% to 85% CH_3CN over 15 min (3-18 min), then gradient from 85% to 95% of CH_3CN over 4 min(18-22 min), then isocratic 95% CH_3CN for 2 min (22-24 min).

Method II for LC-MS (Agilent 6230 LC TOF): Agilent Extend C18 (1.8 µm, 2.1 × 50 mm) analytical column using water-acetonitrile mobile phase with 0.1% formic acid and a flow rate of 0.2 mL/min. Gradient used: isocratic 5% CH_3CN for 3 min (0-3 min), then gradient from 5% to 85% CH_3CN over 15 min (3-18 min), then gradient from 85% to 95% CH_3CN over 4 min (18-22 min), then isocratic 95% CH_3CN for 2 min (22-24 min).

Method III for analytical HPLC (Waters 2695): Phenomenex C18 (5 µm, 2.0 × 150 mm) analytical column using a phosphate buffer (pH 7)-acetonitrile mobile phase with a flow rate of 0.2 mL/min. Gradient used: isocratic 5% CH_3CN for 8 min (0-8 min), then gradient from 5% to 95% CH_3CN over 12 min (8-20 min).

Method IV for semiprep HPLC purification of the conjugates (Waters 2695): Phenomenex C18 (10 µm, 10 × 250 mm) analytical column using water-acetonitrile mobile phase with a flow rate of 5 mL/min. Gradient used: isocratic 5% CH_3CN for 5 min (0-5 min), then gradient from 5% to 95% CH_3CN over 20 min (5-25 min).

II. Conjugation of benzaldehyde and L-cysteine

100 µL stock solution of benzaldehyde (10 mM in phosphate buffer) and 100 µL solution of cysteine (10 mM in phosphate buffer) were mixed with 800 µL phosphate buffer (pH 7, 12% D_2O). The mixture was incubated for 30 min at room temperature and the sample was directly analyzed using ^{1}H-NMR. There was no product formation observed in ^{1}H-NMR peaks. The same reaction mixture was analyzed after 3 hours and <5% product formation was observed. The final concentration of each reagent was 1 mM in the reaction mixture.

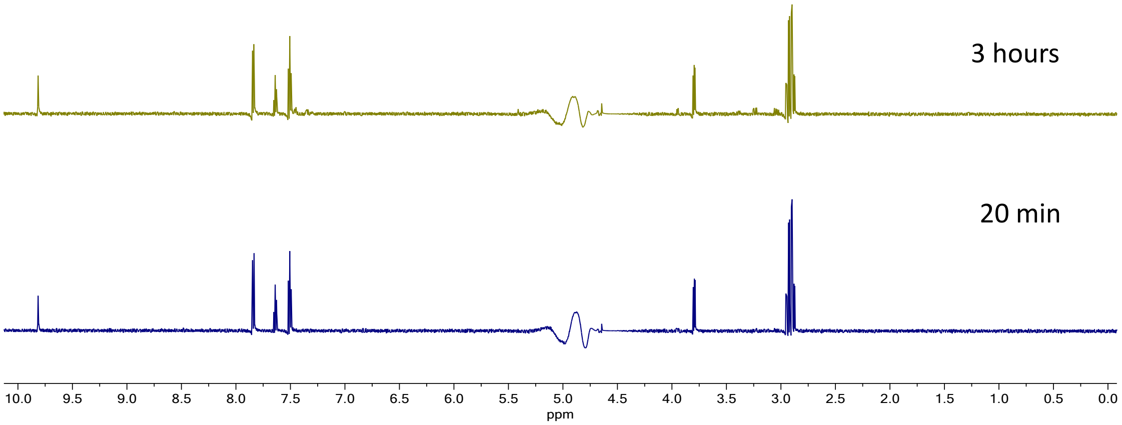

Figure S1. Stacked ^{1}H-NMR spectrum of benzaldehyde and L-cysteine mixture recorded after 20 min (bottom) and 3 hours (top) incubation at room temperature.

III. Conjugation of 2-FPBA and L-cysteine

100 µL stock solution of 2-FPBA (10 mM in phosphate buffer) and 100 µL solution of cysteine (10 mM in phosphate buffer) were mixed with 800 µL of phosphate buffer (pH 7, 12% D_2O). The mixture was incubated for 10 min at room temperature and the sample was directly analyzed using NMR and ESI-MS. The final concentration of each reagent was 1 mM in the reaction mixture for NMR analysis and the mixture was further diluted 20 times to 50 µM for ESI-MS analysis.

IV. NMR studies of the TzB complex formation between 2-FPBA and L-cysteine

The TzB (thiazolidiono boronate) complex (1 mM, 3 mL) of 2-FPBA and L-cysteine was synthesized following the procedure in section III. The solution pH was further adjusted to the desired values using 0.1 N HCl and 0.1 N NaOH solutions. NMR analysis revealed the existence of the two sets of peaks observed at pH 7, which readily interconvert upon pH variation to give predominantly one species at pH below 6 and above 8. For more clear presentation of this phenomenon, the NMR spectra recorded at pH 5.0 and 7.8 are shown below along with the spectrum at pH 7.

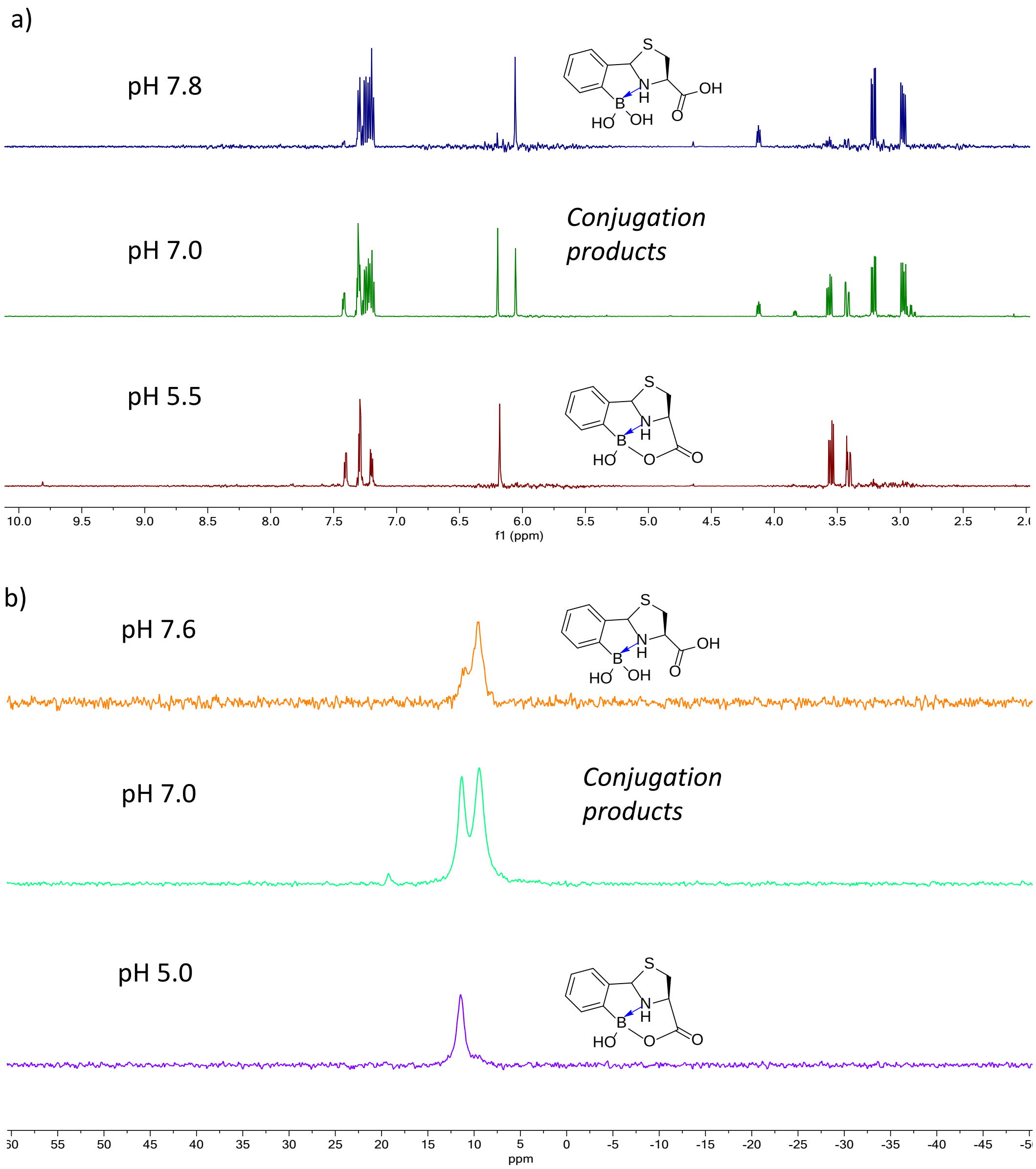

Figure S2. a) ^{1}H-NMR and b) ^{11}B-NMR of the 2-FPBA-cysteine conjugate in different pHs. The data suggest the existence of two interconvertible structures dependent on pH.

a)

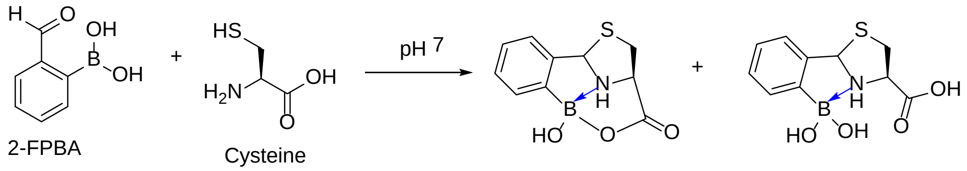

b)

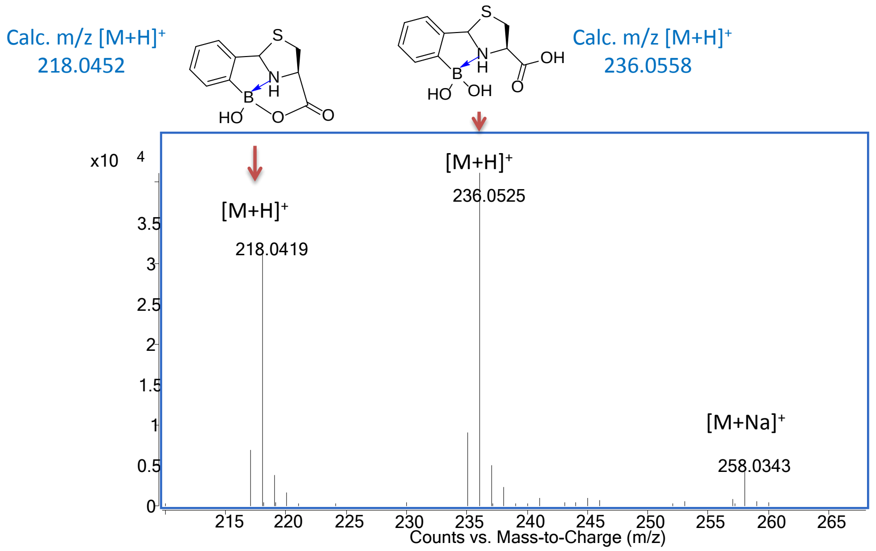

Figure S3. a) Illustration and b) mass-spec proof of the two species generated by conjugation of 2-FPBA with L-cysteine.

V. Conjugation of 2-FPBA and CAL

The conjugation between 2-FPBA and the CAL peptide was analyzed by recording ^{1}H-NMR in 600 MHz at 25 ^{o}C with 64 scans. 100 µL stock solution of 2-FPBA (10 mM in phosphate buffer) and 100 µL solution of CAL (10 mM in phosphate buffer) were mixed with 800 µL of phosphate buffer (pH 7, 12% D_2O). 1 mM of each reagent in the final mixture was incubated for 10 min at room temperature and the sample was immediately analyzed using ^{1}H-NMR. The disappearance of the aldehyde peak confirmed complete conversion. Furthermore, the ^{11}B-NMR sectrum (Figure 2c, main text) was recorded in 500 MHz at 25 ^{o}C with 10,000 scans of conjugate at 5 mM along with 1 mM boric acid as an internal standard. The mixture was diluted to 50 µM and 5 µL was injected for ESI-mass analysis.

^{1}H-NMR (Phosphate buffer pH 7.0 with 10% D_2O) : 7.34-7.21 (m, 3H), 7.16-7.15 (d, *J* = 5.4, 1H), 6.03 (s, 1H), 4.32-4.28 (q, *J* = 4.8, 1H), 4.23 (b, 1H), 4.09-4.06 (t, *J* = 4.6, 1H), 3.26-3.13 (m, 2H), 1.59-1.46 (m, 3H), 1.28-1.27 (d, *J* = 4.7, 2H), 0.83-0.77 (dd, *J* = 4.7, *J* = 9.6, 6H).

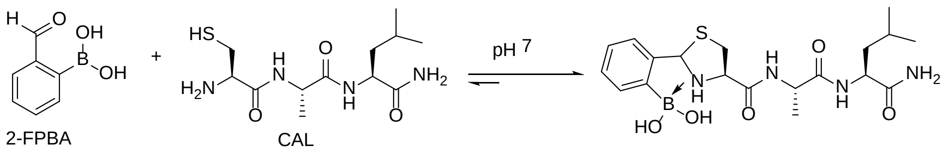

a)

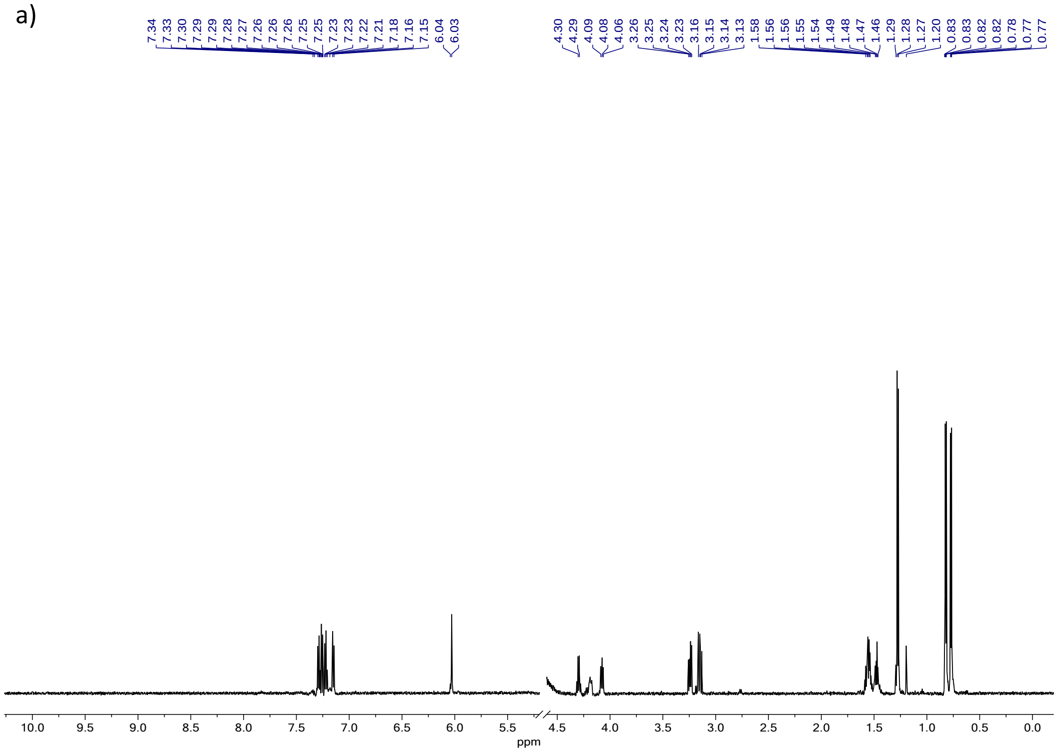

b)

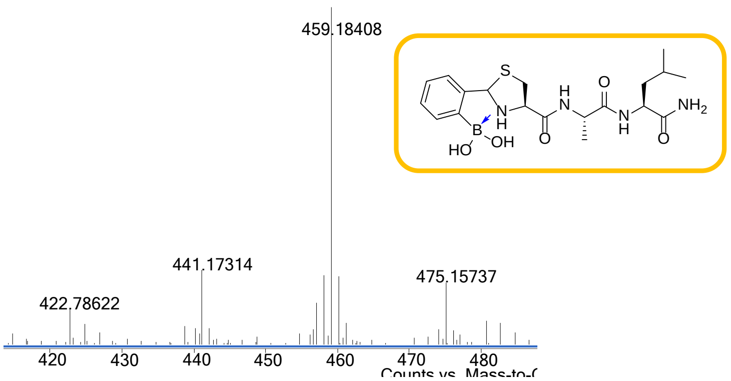

Figure S4. a) ^{1}H-NMR and b) mass-spec characterization of the 2-FPBA-CAL TzB conjugate.

VI. Kinetics study of the TzB complex formation between 2-FPBA and CAL

The kinetics of the conjugation reaction between the CAL peptide and 2-FPBA were studied by recording UV-Vis absorption with a Nanodrop UV-vis spectrometer. The data were collected after blank subtraction using phosphate buffer (pH 7). The reaction was performed in a quartz cuvette (10 mm path length; total volume ~ 2 mL) using phosphate buffer (10 mM, pH 7) pre-equilibrated at room temperature. 1 mL PBS buffer was pipetted into the cuvette, followed by the addition of 1 µL of the 2-FPBA stock (10 mM) in the buffer. The solution was mixed rapidly and the spectrum of 2-FPBA was recorded. Further, 1 µL of the CAL stock (10 mM) was added into the 2-FPBA (10 µM) solution in the cuvette. After quick mixing, UV-vis data of the reaction mixture were collected on 10 second intervals. A quick decrease of the absorption maxima at ~ 254 nm was observed upon product formation. The experiment was repeated three times and consistent results were obtained. The kinetic data were fitted according to a second order mechanism with the two reactants at equal concentrations.

The conjugation reaction at 10 µM was found to go to essentially 100% completion as the end spectrum at 10 µM gave the same profile as that obtained at 100 µM, and complete conjugation at 100 µM was confirmed by ^{1}H-NMR analysis (Figure S5b).

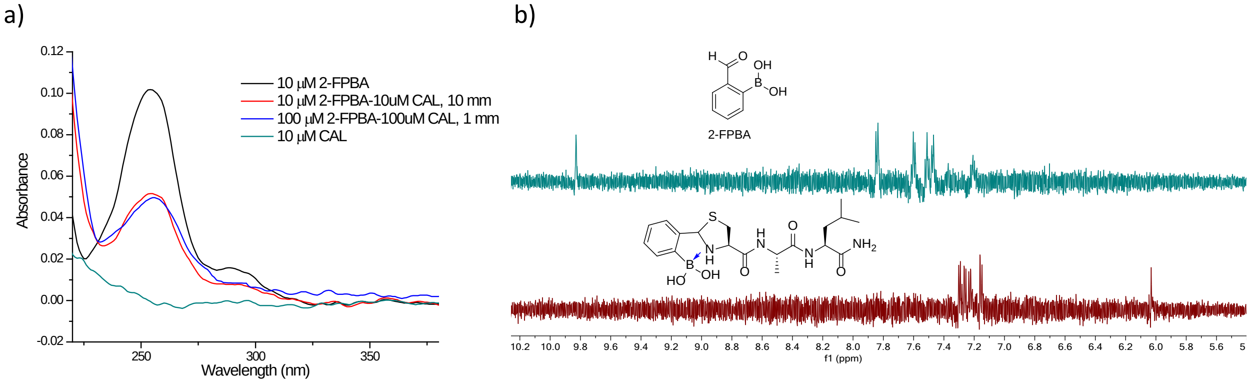

Figure S5. a) UV-vis profile overlay of 2-FPBA-CAL complex at 10 and 100 µM to confirm the complete conjugation at 10 µM. b) ^{1}H-NMR data (600 MHz at 25 ^{o}C with 160 scans) showing complete conjugation of 2-FPBA and CAL at 100 µM.

VII. Protein labelling experiments

50 µL stock solutions of 2-FPBA-NBD and Cys-VHP were mixed (10 µM final concentrations) and incubated for 30 minutes in phosphate buffer (pH 7) at room temperature. In HPLC analysis using **Method III** (page S2), the mixture gave a peak that eluted about 0.5 min later than 2-FPBA-NBD. To confirm the observed peak is the product instead of starting material, an additional experiment was done with one equivalent excess of 2-FPBA-NBD. The LC trace of the 2:1 mixture gave two peaks, indicating 2-FPBA-NBD and its conjugate with VHP can be separated with our LC gradient. The fact that a single peak was observed for the 1:1 mixture indicates the conjugation between 2-FPBA-NBD and Cys-VHP went to completion under our experimental conditions. The product formation is further supported by the mass-spec data, which shows the expected molecular ions of the labelled VHP.

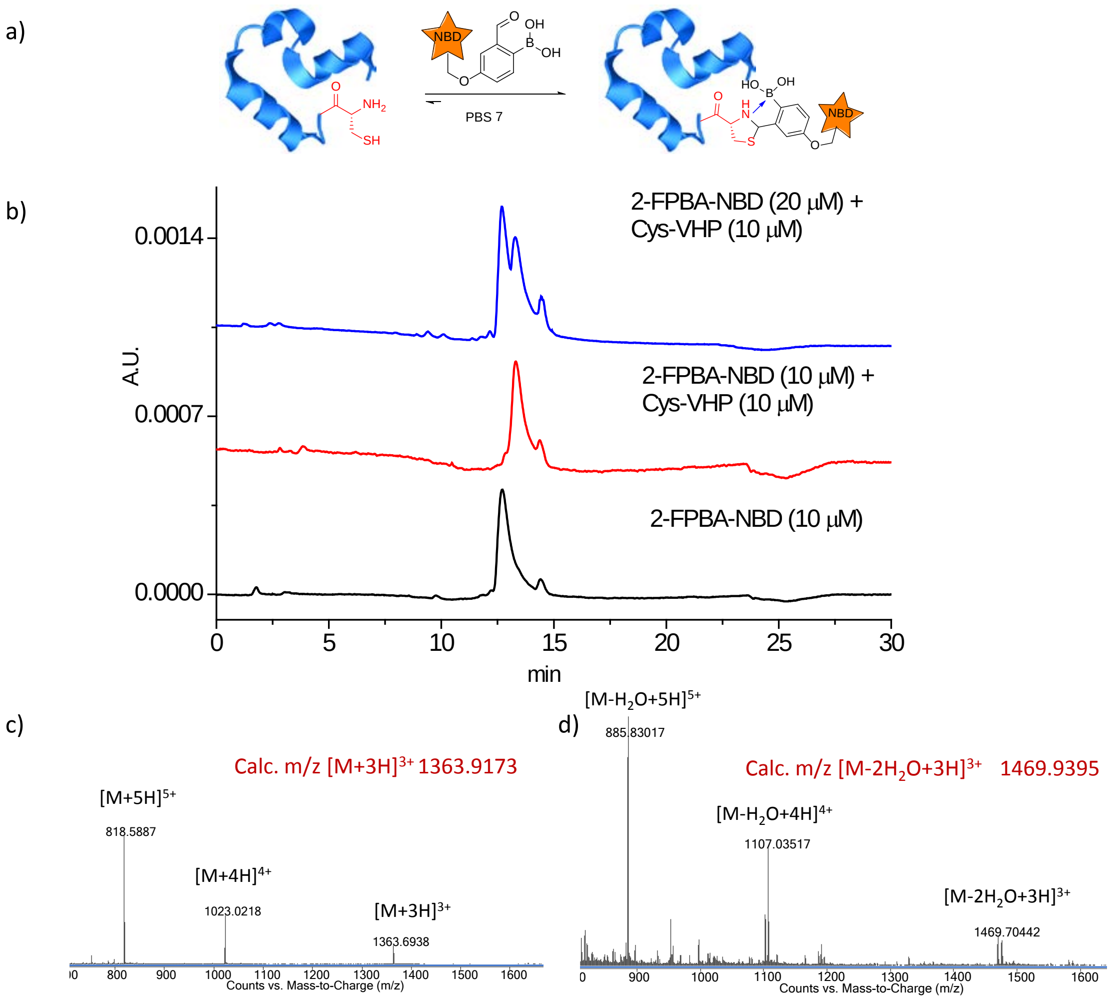

Figure S6. a) Scheme for N-terminal protein labeling through TzB complex formation. b) HPLC traces (Method III) for 2-FPBA-NBD alone and its mixtures with Cys-VHP. A small impurity (~ 6%) at 14.4 min appeared due to the oxidation of boronic acid to phenol, which was confirmed by mass spectrum analysis. c) Mass spec data of Cys-VHP before and d) after conjugation with 2-FPBA-NBD. Water loss is commonly seen in the mass-spec results of boronic acid-presenting compounds.[1]

VIII. Purification and storage of TzB complexes

Although the TzB adducts are sensitive to acidic medium, they can be readily purified under neutral conditions. The TzB complexes of the peptide CAL and model protein VHP were purified via HPLC with neutral eluent (water-acetonitrile as mobile phase with no acid) using **Method IV** on Page S2. The purified materials were dried through lyophilization and isolated yields were calculated.

Purification and storage of the TzB complex of CAL

0.75 mg (0.005 mmol) of 2-FPBA and 2 mg of CAL (0.005 mmol) peptide was incubated for 5 min in phosphate buffer (pH 7). Then the conjugate product was purified using **Method IV** (page S2). As indicated by Figure 2 of the main text, this conjugation reaction readily goes to completion with no side product. This is validated by the prep HPLC trace shown below, which displays a single peak (Figure S7a). The collected peak was directly lyophilized to afford white powder with 86% yield (yield calculated by weight). The purity and identity of the conjugate were confirmed by ^{1}H-NMR and ESI-MS (Figure S7b, c).

The stability of the TzB complex during storage in solution was assessed by dissolving 0.85 mg of the 2-FPBA-CAL conjugate in a phosphate buffer (pH 7, 5 mL). The solution was stored in a 4 °C fridge. Stability of the TzB complex was assessed by recording ^{1}H-NMR over time (Figure S7c).

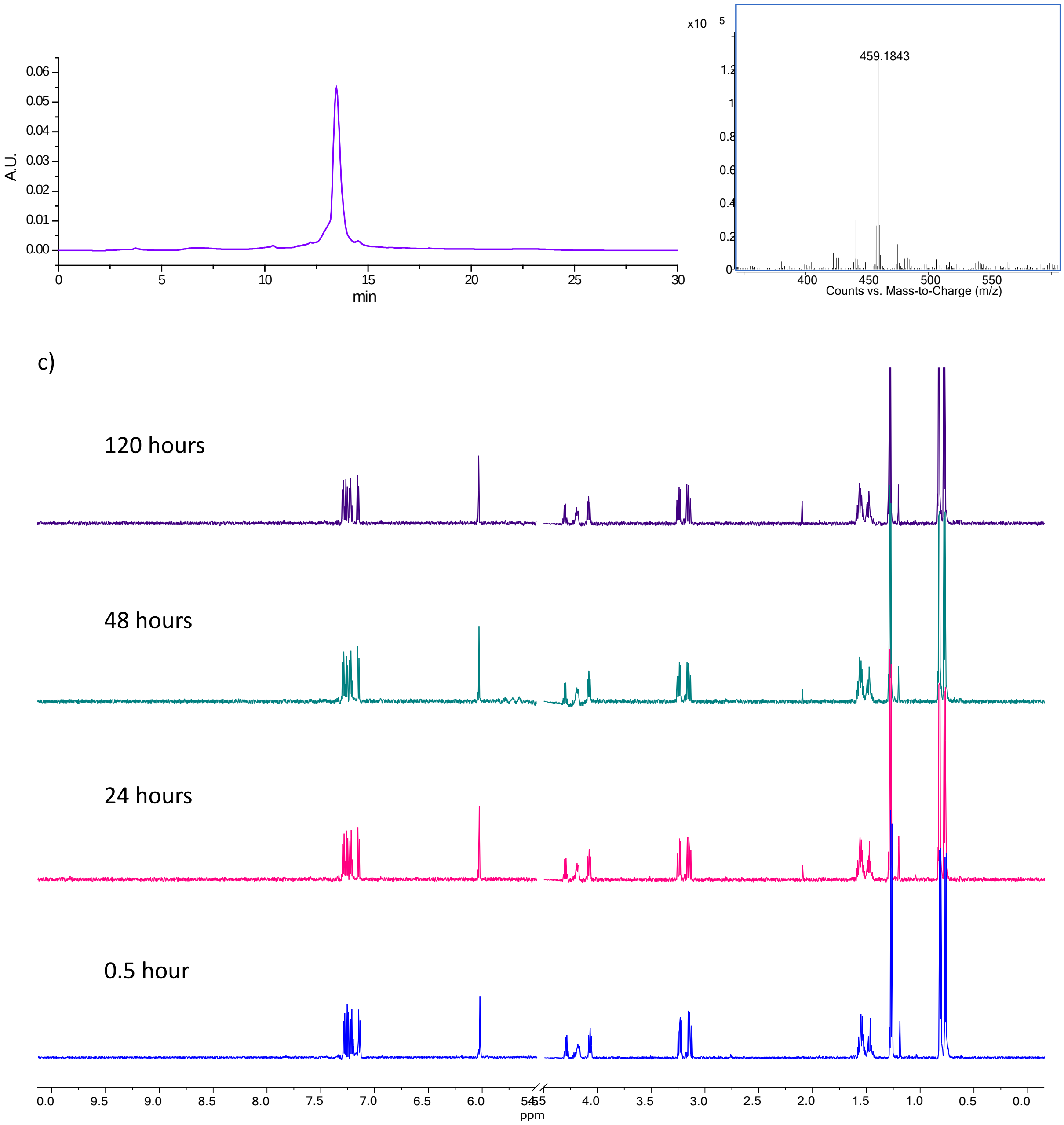

Figure S7. a) Prep HPLC trace of the 2-FPBA-CAL TzB conjugate with neutral eluent. b) ESI-MS of the isolated 2-FPBA-CAL TzB conjugate confirming the identity of the compound. c) Assessing stability of the 2-FPBA-CAL TzB conjugate during storage in neutral buffer at 4 ^{o}C over time. The stacked ^{1}H-NMR spectra shows little decomposition of the compound even after 5 days.

Purification of the TzB complex of VHP

Cys-VHP (200 µM, 100 µL) and 2-FPBA-NBD (220 µM, 100 µL) were mixed together and incubated for 10 min. The mixture was then purified under neutral conditions using **Method IV** as specified on page S2. The product peak was collected and lyophilized to afford an orange powder (78% yield). The yield was measured using UV-vis absorption of NBD at 495 nm. To confirm purity and identity, a 20 µM solution was injected for LC-MS analysis using **Method I** (page S2). The major peak (~95%) at 13.94 min gives the mass of the desired product. A small peak (~5%) at 13.42 min was also found to give the desired mass, indicating that this small peak corresponds to a minor conformational isomer of the TzB complex that likely originates from the use of neutral eluents.

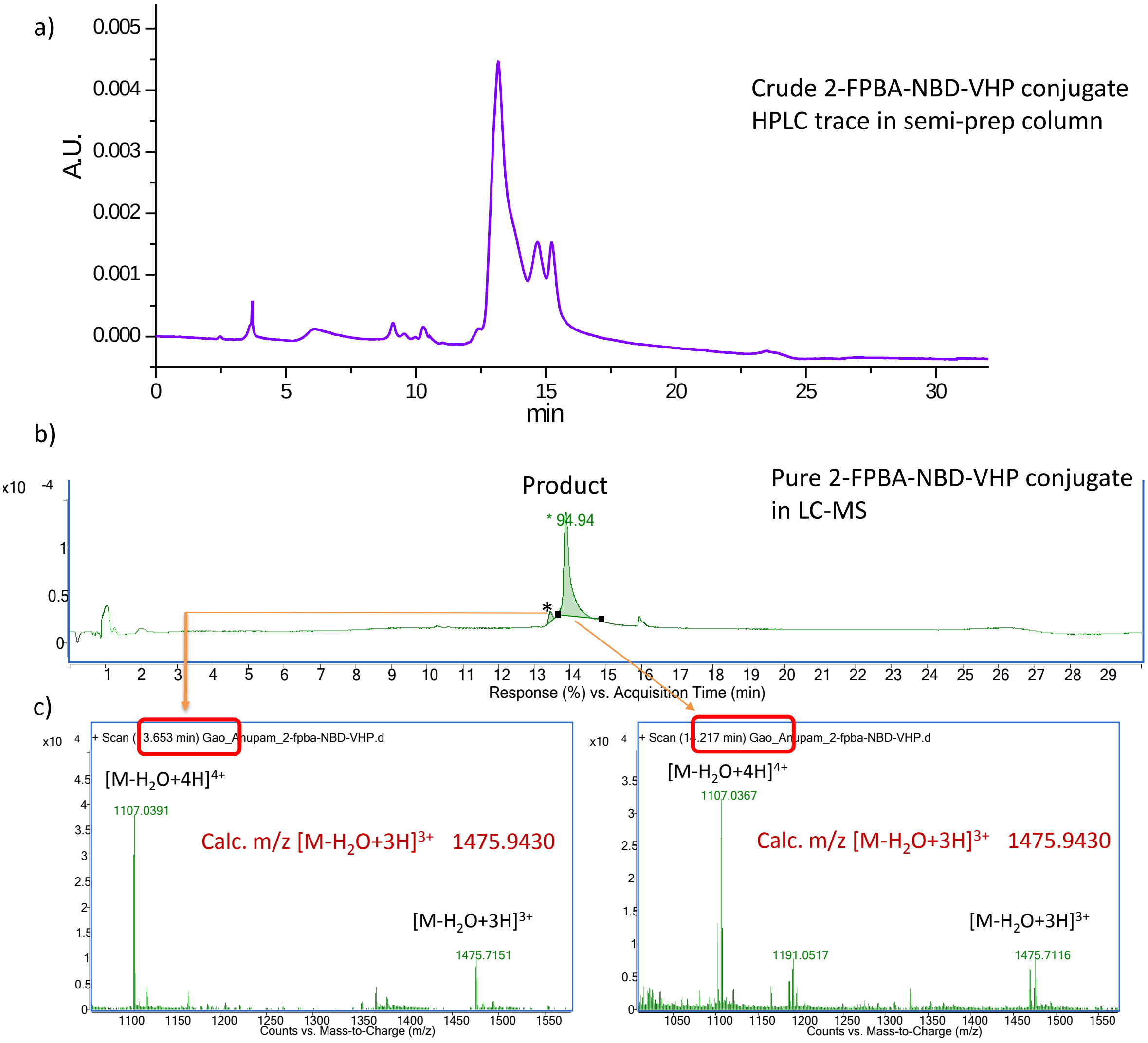

Figure S8. a) Prep HPLC trace of the TzB complex of Cys-VHP and 2-FPBA-NBD. b) LC and c) MS verification of the purity and idendentiy of the isolated product; masses of the peaks in the LC trace are shown with the retention times highlighted in c). Water loss is commonly seen in the mass-spec results of boronic acid-presenting compounds.[1]

IX. Comparing the stability of the 2-FPBA-CAL TzB complex and the salicylaldehyde-CAL thiazolidine conjugate

The 2-FPBA-CAL TzB conjugate was prepared following the procedure described in section V and used for analysis without purification. For NMR analysis, 10% D_2O was added into the sample buffer to give the final peptide concentration of 0.4 mM. The ^{1}H-NMR spectrum of the sample was taken after 10 min incubation and again after 10 hours at room temperature. No detectable product dissociation or decomposition was observed. For comparison, the salicylaldehyde-CAL thiazolidine conjugate (see section XV for details of synthesis) was dissolved in neutral phosphate buffer with 10% D_2O (final peptide concentration is 1.0 mM). The sample was analyzed by ^{1}H-NMR after 20 min and 10 hr incubation at room temperature. Approximately 25% dissociation of salicylaldehyde-CAL conjugate was observed at the 10 hr time point.

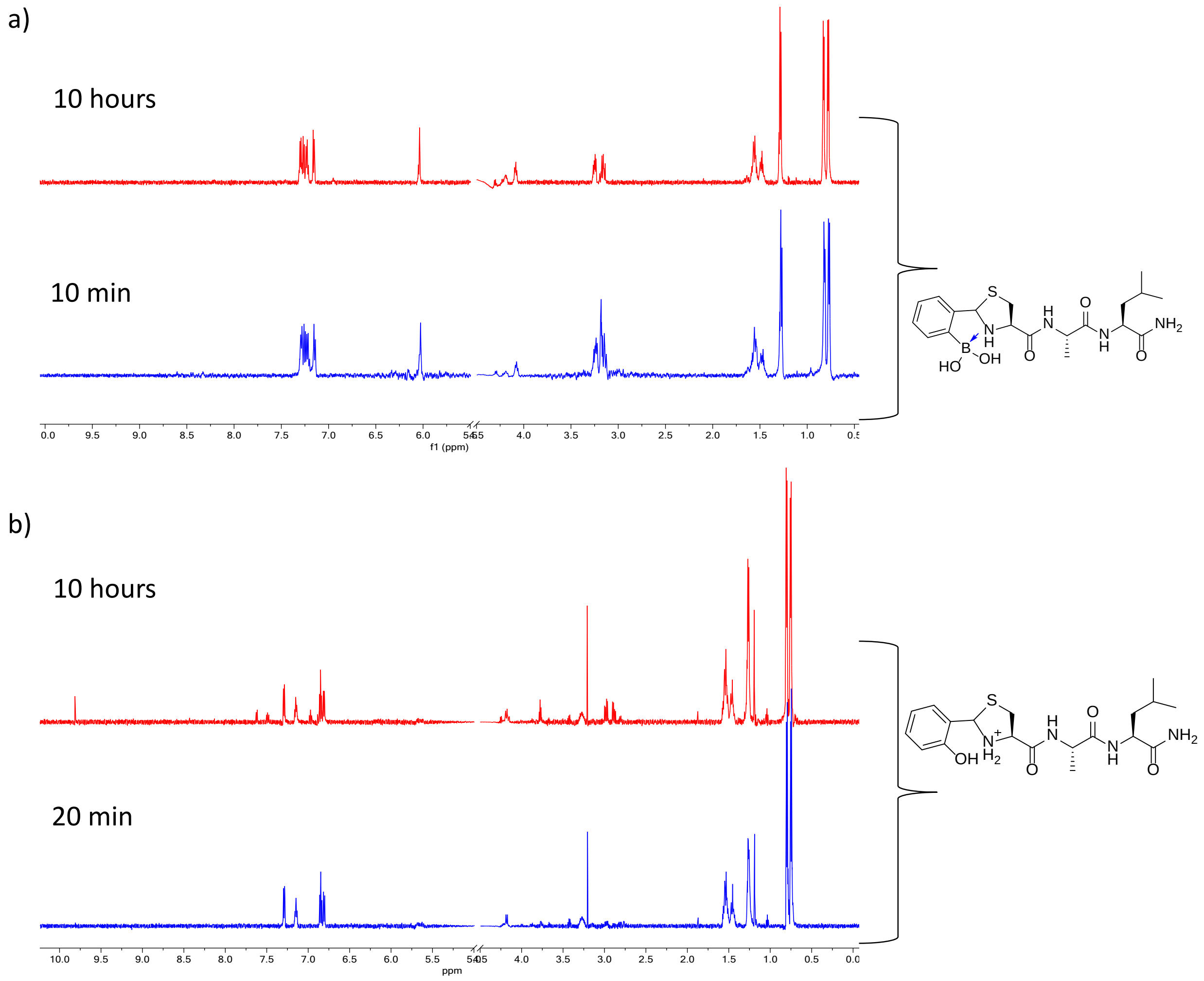

Figure S9. Comparative analysis of the a) 2-FPBA-CAL TzB conjugate and b) salicylaldehyde-CAL thiazolidine conjugate for degradation over time.

X. Small molecule inhibition experiments for TzB complex formation

100 µL stock solution of the CAL peptide (6 mM) in a phosphate buffer (pH 7.0) was diluted with 300 µL phosphate buffer containing 10% D_2O. The solution was mixed with 100 µL stock solution of lysine (90 mM), glucose (30 mM), glutathione (30 mM), serine (30 mM) or cystine (6 mM). The pH of final mixtures (600 µL total volume) was tuned to 7.0 using 0.5 N NaOH and/or 0.5 N HCl. Additionally, 100 µL stock solution of 2-FPBA (6 mM) was added to each individual mixture. The inhibition effect on product formation was examined using ^{1}H-NMR. The data show that the signature resonances of the TzB complex, including the aromatic region and the thiazolidine resonance at 6 ppm, remain unchanged with the addition of the potential small molecule inhibitors. The spectra were obtained using the 600 MHz Avance NMR spectrometer at 25 ^{o}C with 64 scans. The data were processed using MestReNova 10.0.

a)

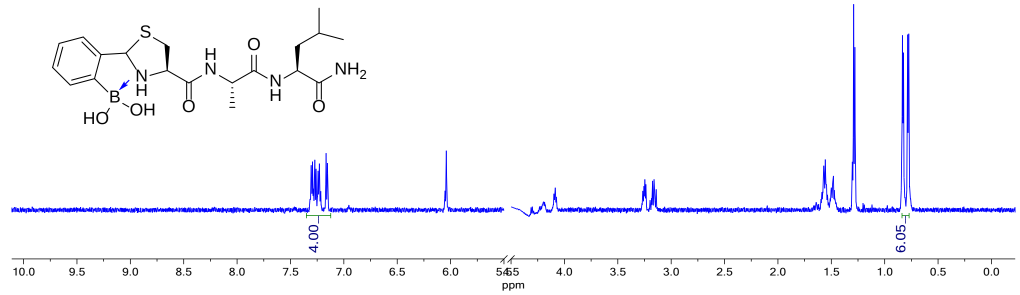
S
H
N
O
N
H
B
HO
OH
N
H
NH2
O
O
4.00
6.05
ppm

b)

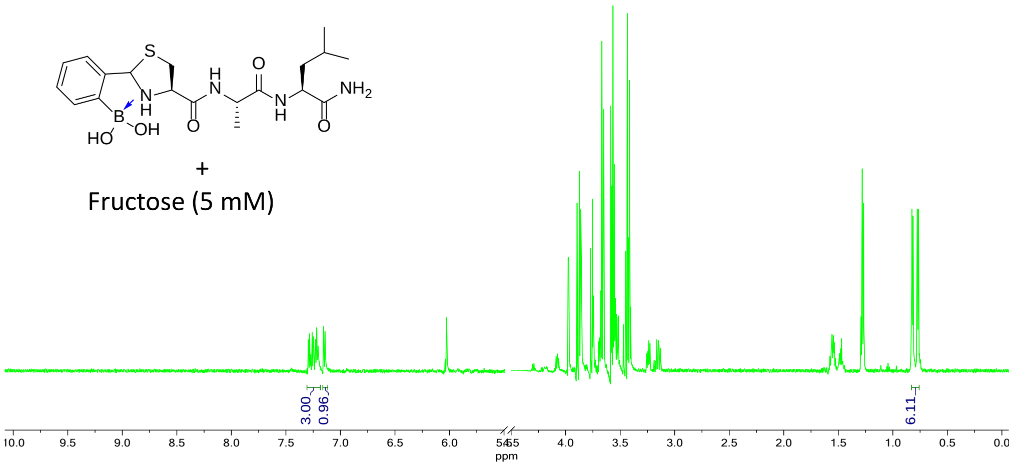
+
Fructose (5 mM)
3.00
0.96
6.11
ppm

c)

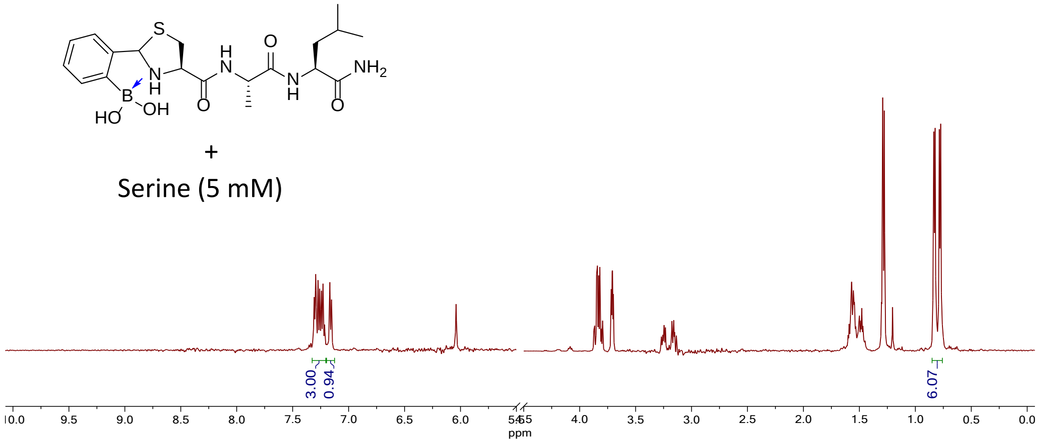
+
Serine (5 mM)
3.00
0.94
6.07
ppm

d)

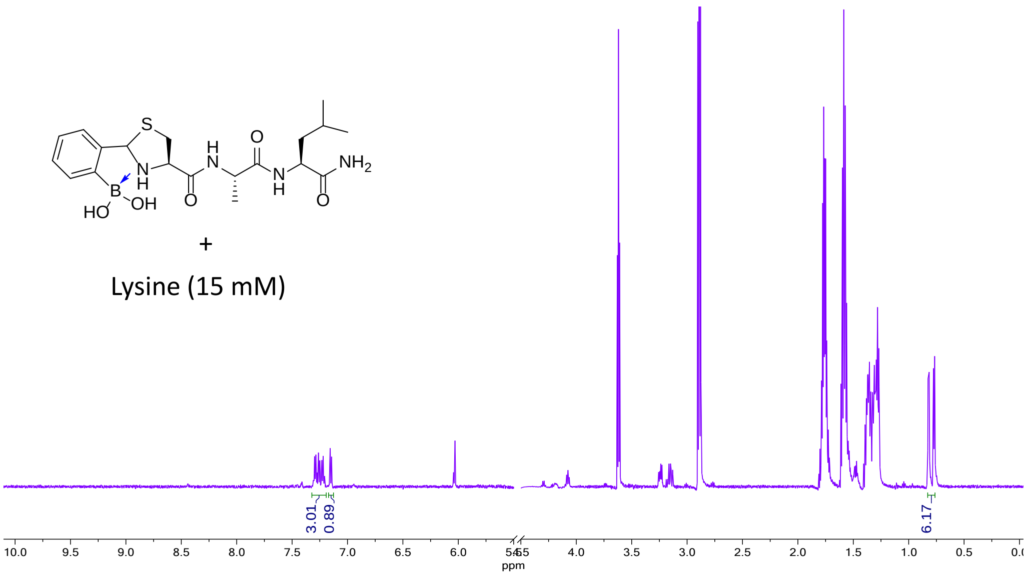

e)

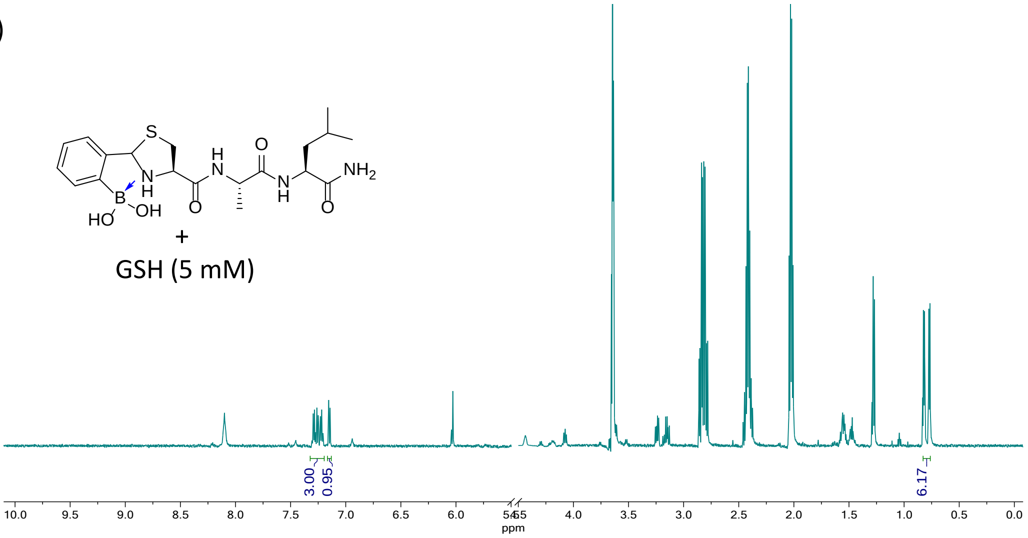

f)

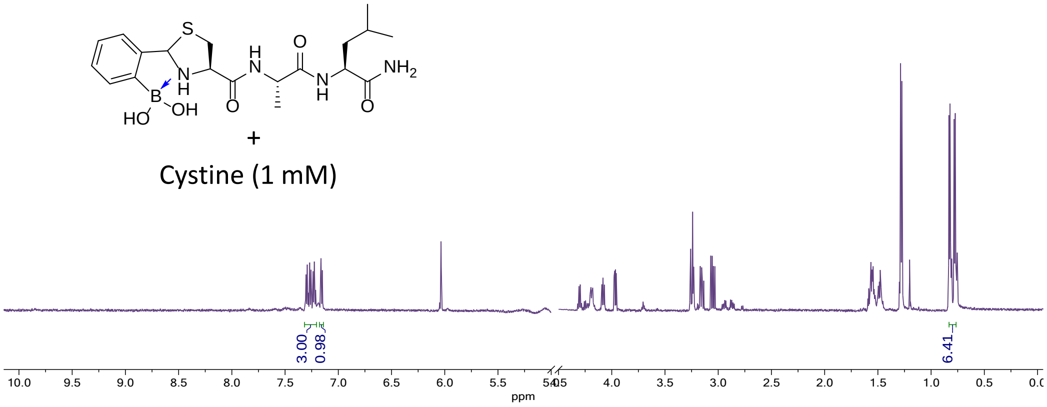

Figure S10. TzB complex formation in presence of abundant biomolecules. a) Control reaction; b) fructose (5 mM); c) serine (5 mM); d) lysine (15 mM); e) glutathione (5 mM); f) cystine (1 mM).

XI. Testing the reactivity of 2-FPBA toward a spatially preorganized Cys-Lys pair in α-helix

An Ala-rich helical peptide (H1: WAEAAAKCAAAKEAAAKA-NH_2) was synthesized (see section XVI for details) to display a Cys-Lys pair at i and i+4 positions. The sequence was chosen based on the stabilized peptide helices reported by Marqusee and Baldwin (PNAS, 1987, **84**, 8898-902). ^{1}H-NMR spetra were taken with the peptide H1 alone (1 mM, pH 7 phosphate buffer with 10% D_2O), as well as in mixture with equimolar 2-FPBA. The mixture was incubated for 1 hour before NMR analysis. The pH of all samples was tuned to 7.0 using 0.1 N HCl and 0.1 N NaOH. The ^{1}H-NMR spectrum of the mixture is essentially the addition of those of the pure components, indicating no reaction between 2-FPBA and the Cys-Lys pair in a helix.

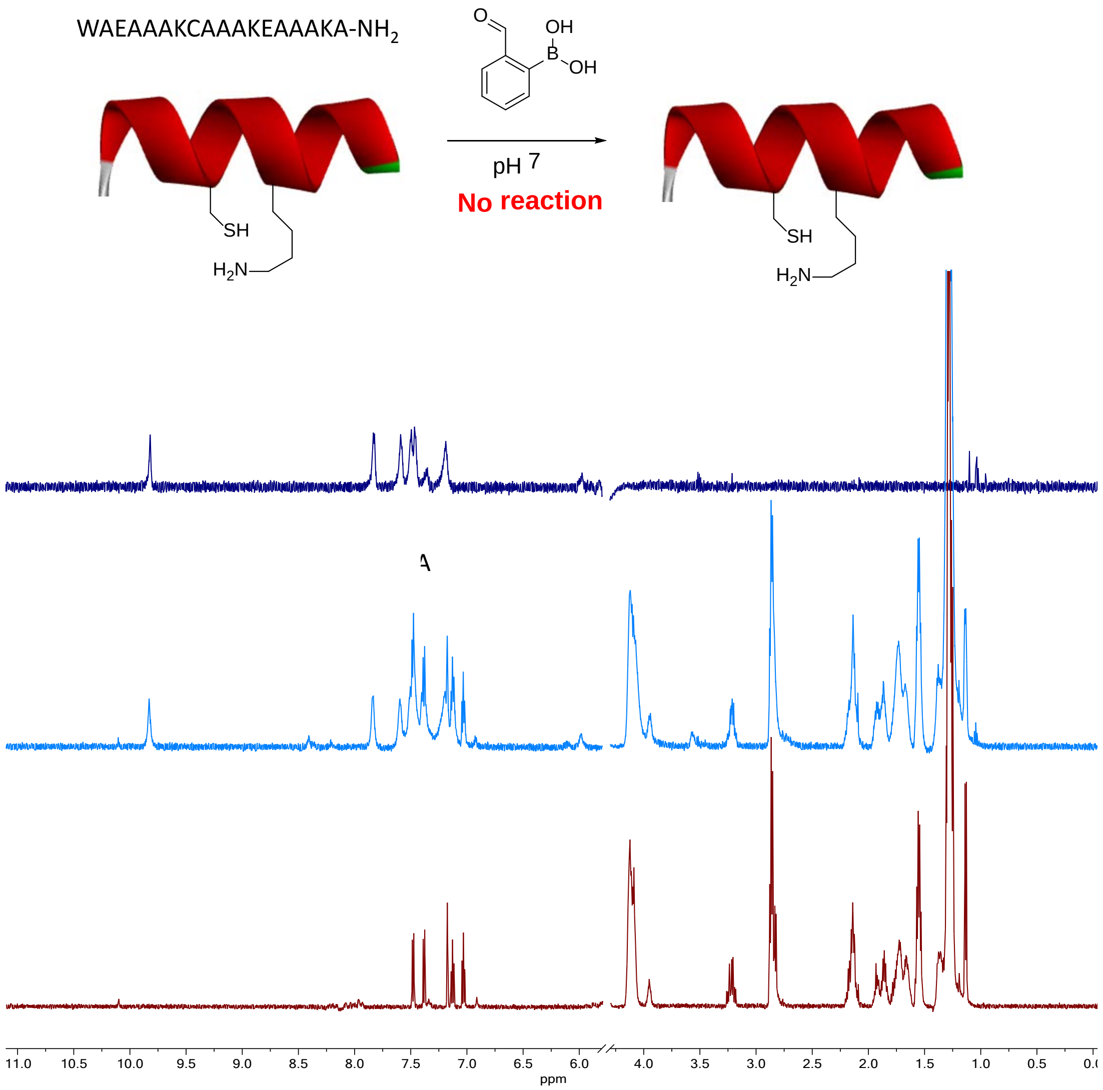

Figure S11. a) Cartoon representation of reaction between 2-FPBA and a helical peptide bearing a Cys-Lys pair at i and i+4 positions. b) Stacked ^{1}H-NMR spectra of the helical peptide H1 alone (bottom), 2-FPBA alone (top) and the mixture of H1 and 2-FPBA (middle).

XII. L-cysteine exchange with a TzB conjugate

The exchange of L-cysteine with the 2-FPBA-CAL TzB conjugate was monitored via ^{1}H-NMR. 1 mM TzB conjugate of 2-FPBA-CAL was prepared following the previously explained protocol (section V) in neutral buffer. Complete conjugation was confirmed by ^{1}H-NMR and the spectrum was considered as the starting point (0 min). 10 μL of an L-cysteine stock solution (60 mM, phosphate buffer pH = 7) was then added to the TzB conjugate in an NMR tube and was mixed homogeneously. Cysteine exchange with TzB conjugate was monitored by recording ^{1}H-NMR over time. The percentage of exchange was calculated by integrating the thiazolidine peak (6.04 ppm) of 2-FPBA-CAL versus the thiazolidine peaks (6.06 and 6.2 ppm) of 2-FPBA-cysteine. The spectra were obtained using a 500 MHz Avance NMR spectrometer at 25 °C with 64 scans. The data were processed and stacked using MestReNova 10.0.

a)

b)

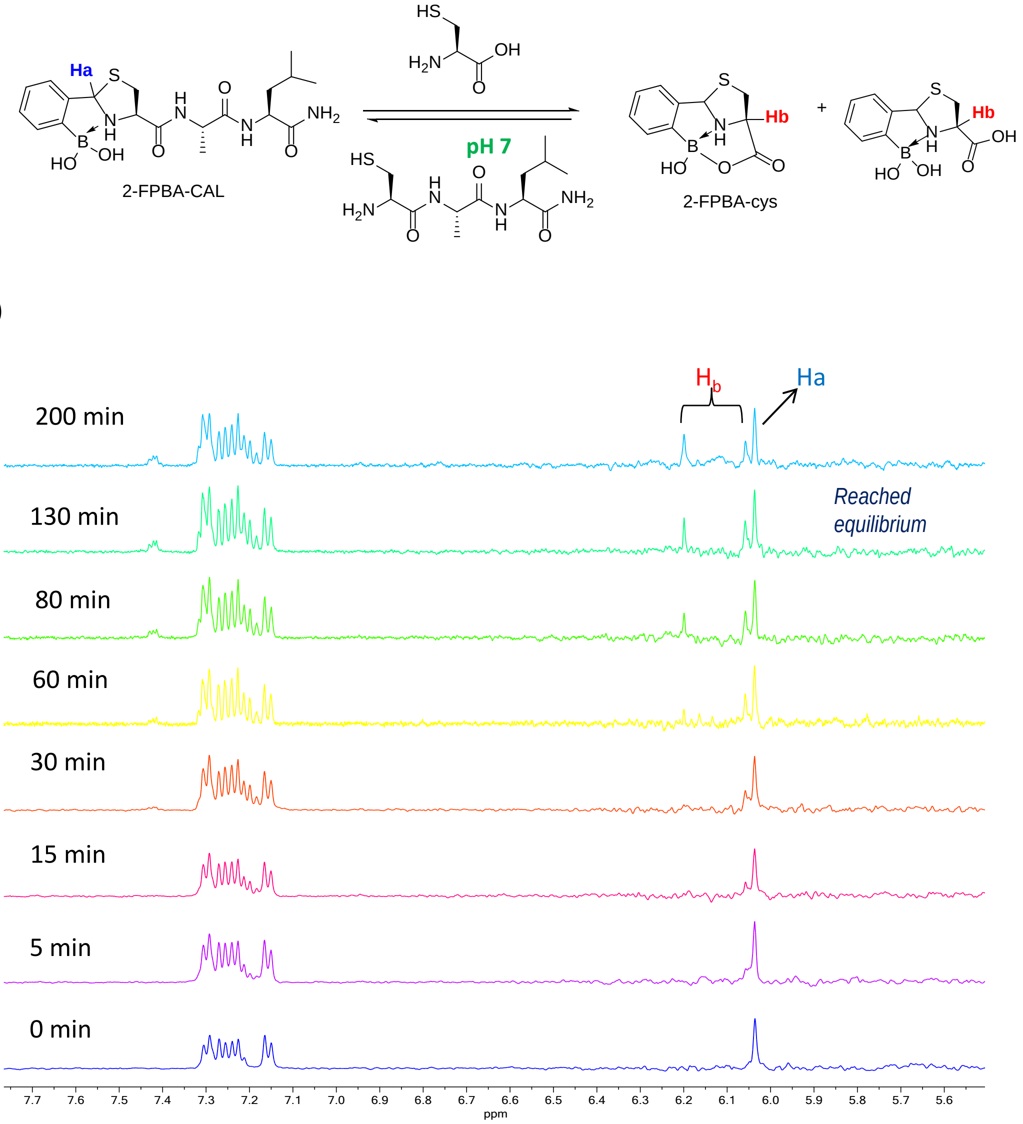

Figure S12. Exchange of L-cysteine (1 mM) with the TzB conjugate of 2-FPBA and CAL in a neutral buffer. a) Reaction scheme of exchange and b) stacked ^{1}H-NMR spectra over time.

XIII. Dissociation of a TzB conjugate in acidic pH

A mixture of 2-FPBA (0.4 mM) and CAL (0.4 mM) was prepared in phosphate buffer (pH 7, 5 mL) with 10% D_2O. Then 600 μL aliquots of the sample were adjusted to different pHs (2.5, 3.2, 4, 4.5, 5, 5.5, 6) using 0.5 N HCl and/or 0.5 N NaOH. ^{1}H-NMR was recorded shortly after the pH is adjusted (<10 min) and then again at 20 hrs. The spectra were obtained using a 600 MHz Avance NMR spectrometer at 25 ^{o}C with 96 scans. The data were processed and stacked using MestReNova. The ratio of product and starting material was calculated by integrating the peak at ~ 6 ppm for the TzB complex and the peak at ~ 9.8 ppm for the free aldehyde.

a)

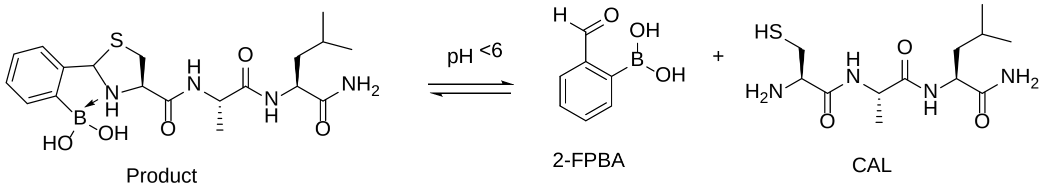

b)

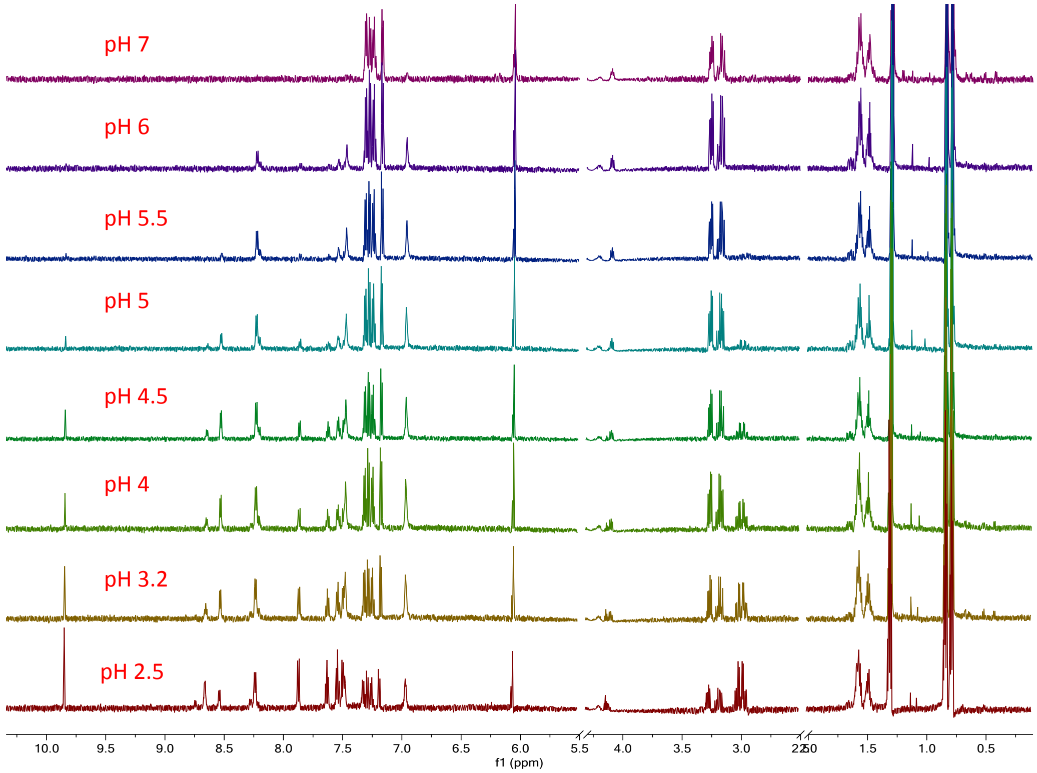

Figure S13. a) Scheme and b) ^{1}H-NMR data for the dissociation of a TzB complex under acidic conditions.

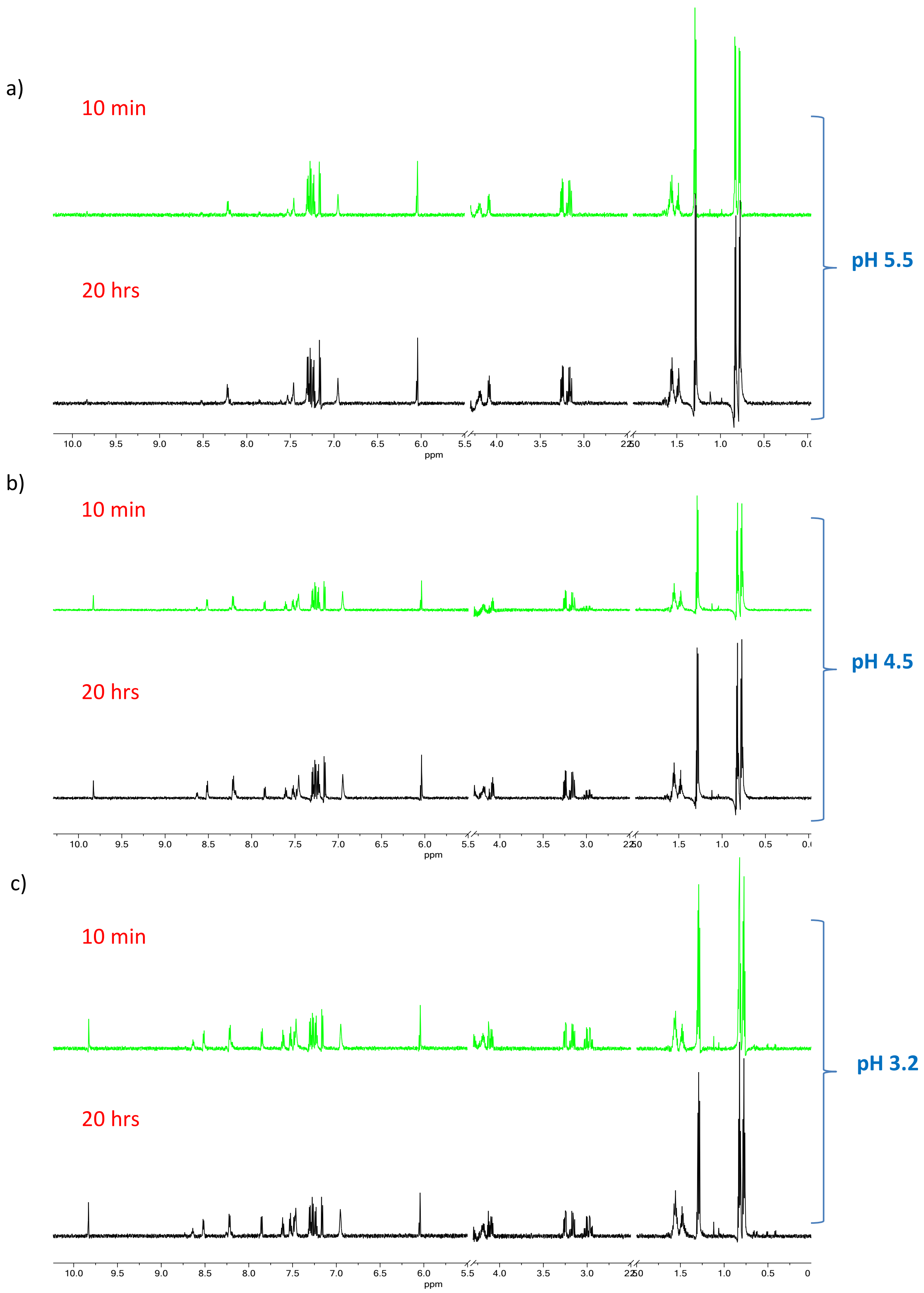

Figure S14. Dissociation studies of the 2-FPBA-CAL TzB conjugate at pH a) 5.5, b) 4.5, c) 3.2 after 20 hours.

XIV. Crystallographic information for 2-FPBA-cysteine thiazolidino boronate conjugate

2-FPBA (15 mg, 0.1 mmol) and L-cysteine (12.1 mg, 0.1 mmol) were dissolved in 1 mL of 1:1 (MeOH:water) at pH 7.0. The pH of the final solution was adjusted to pH ~ 7.0 using 0.01 N NaOH. The solution was then subjected to slow evaporation in a 5 mL glass test tube. After 24 hours, rod shaped crystal growth was observed.

Table 1. Crystal data and structure refinement for 2-FPBA-cysteine complex.

Identification code	$C_{10}H_{10}BNO_3S(H_2O)$	
Empirical formula	C_{10} H_{12} B N O_4 S	
Formula weight	253.08	
Temperature	100(2) K	
Wavelength	1.54178 Å	
Crystal system	Triclinic	
Space group	P1	
Unit cell dimensions	a = 5.8323(4) Å	α= 97.280(2)°.
	b = 6.9890(4) Å	β= 98.850(2)°.
	c = 13.7431(9) Å	γ = 91.576(2)°.
Volume	548.43(6) $Å^3$	
Z	2	
Density (calculated)	1.533 Mg/m^3	
Absorption coefficient	2.665 mm^{-1}	
F(000)	264	
Crystal size	0.360 x 0.180 x 0.080 mm^3	
Theta range for data collection	3.283 to 66.850°.	
Index ranges	-6<=h<=6, -8<=k<=8, -16<=l<=16	
Reflections collected	12477	
Independent reflections	3748 [R(int) = 0.0288]	
Completeness to theta = 66.850°	99.5 %	
Absorption correction	Semi-empirical from equivalents	
Max. and min. transmission	0.7528 and 0.6232	
Refinement method	Full-matrix least-squares on F^2	
Data / restraints / parameters	3748 / 11 / 331	
Goodness-of-fit on F^2	1.033	
Final R indices [I>2sigma(I)]	R1 = 0.0260, wR2 = 0.0701	
R indices (all data)	R1 = 0.0261, wR2 = 0.0703	
Absolute structure parameter	0.012(7)	

Extinction coefficient	na
Largest diff. peak and hole	0.207 and -0.175 e.Å^{-3}

Table 2. Atomic coordinates (x 10^4) and equivalent isotropic displacement parameters (Å^{2}x 10^3) for $C_{10}H_{10}BNO_3S(H_2O)$. U(eq) is defined as one third of the trace of the orthogonalized U^{ij} tensor.

	x	y	z	U(eq)
S(1)	7862(1)	233(1)	8393(1)	21(1)
O(1)	3412(4)	-2172(3)	6058(2)	23(1)
O(2)	3143(3)	991(3)	6495(1)	18(1)
O(3)	4016(4)	3750(3)	5774(1)	17(1)
N(1)	7205(4)	2000(3)	6774(2)	16(1)
B(1)	4574(5)	2868(4)	6651(2)	16(1)
C(1)	8258(5)	-1182(4)	7240(2)	22(1)
C(2)	6918(5)	-129(4)	6440(2)	19(1)
C(3)	4313(5)	-547(4)	6310(2)	18(1)
C(4)	4763(5)	4199(4)	7698(2)	16(1)
C(5)	3293(5)	5567(4)	8047(2)	19(1)
C(6)	3915(6)	6692(4)	8964(2)	24(1)
C(7)	6057(6)	6493(5)	9542(2)	26(1)
C(8)	7563(6)	5161(4)	9204(2)	22(1)
C(9)	6895(5)	4022(4)	8291(2)	18(1)
C(10)	8278(5)	2455(4)	7855(2)	18(1)
S(2)	2084(1)	5906(1)	1626(1)	17(1)
O(4)	6635(3)	4811(3)	3882(1)	18(1)
O(5)	6950(3)	7789(3)	3484(1)	13(1)
O(6)	6118(3)	10859(3)	4228(1)	14(1)
N(2)	2890(4)	8513(3)	3238(2)	13(1)
B(2)	5508(5)	9534(4)	3340(2)	13(1)
C(11)	1758(5)	5071(4)	2797(2)	17(1)
C(12)	3161(5)	6576(4)	3568(2)	14(1)
C(13)	5770(5)	6290(4)	3660(2)	14(1)
C(14)	5312(5)	10323(4)	2289(2)	14(1)
C(15)	6812(5)	11531(4)	1926(2)	17(1)

C(16)	6167(5)	12174(4)	1006(2)	21(1)
C(17)	3992(6)	11640(4)	455(2)	23(1)
C(18)	2454(5)	10439(4)	804(2)	19(1)
C(19)	3159(5)	9783(4)	1711(2)	15(1)
C(20)	1759(5)	8390(4)	2167(2)	15(1)
O(7S)	9136(4)	4841(4)	5827(2)	30(1)
O(8S)	884(4)	11497(3)	4202(2)	19(1)

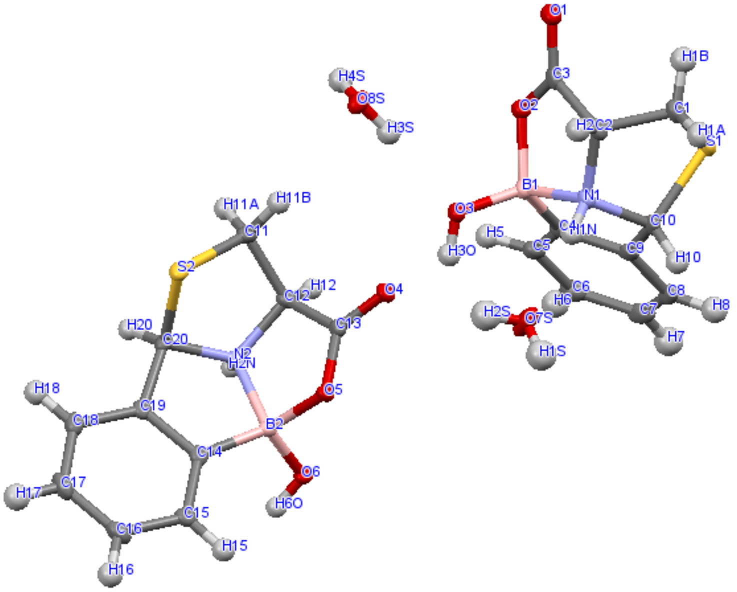

Figure S15. ORTEP diagram of 2-FPBA-cysteine thiazolidino boronate conjugate. Ellipsoids are displayed at 50% probability. Two molecules are present in the asymmetric unit with two water molecules as the co-crystal.

XV. Synthesis of thiazolidine conjugate between salicylaldehyde and the CAL peptide

2 M salicylaldehyde in methanol (0.1 mL, 4 eq) and 10 mM CAL peptide in a phosphate buffer (pH 5.2, 0.5 mL, 1 eq) was mixed together. After 5 hours, the reaction mixture was washed with diethyl ether (4 × 0.4 mL) and the reaction mixture was lyophilized. The powder lyophilized product was dissolved in 1 mL nano pure water and the pH of the solution was tuned to 7 using 0.5 N NaOH or 0.5 N HCl. Clean product formation was confirmed by mass and the disappearance of aldehyde chemical shift of salicylaldehyde in ^{1}H-NMR. The stock thiazolidine solution was further diluted to 0.4 mM and the stability of the product was monitored after 10 hours via ^{1}H-NMR, as explained in section IX and shown in Figure S9b.

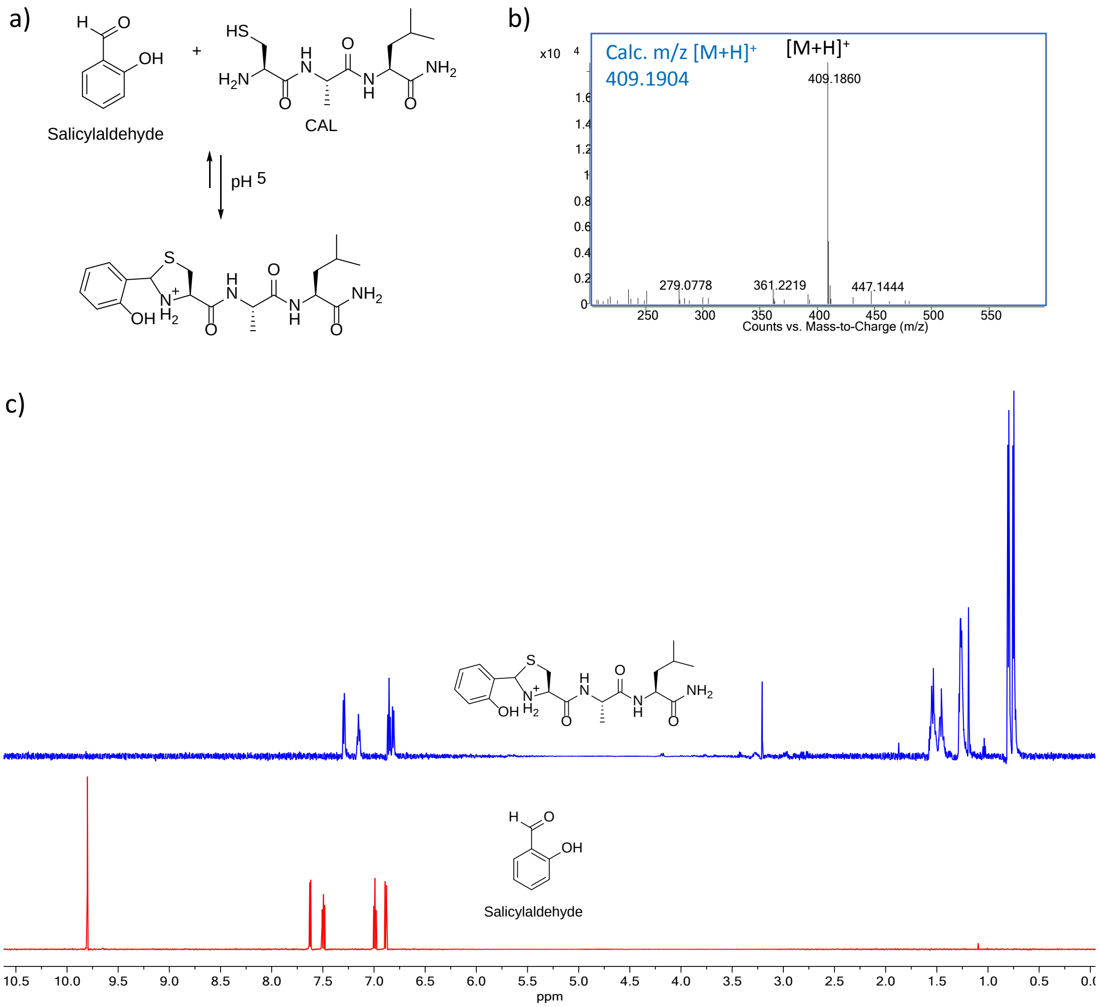

Figure S16. a) Reaction scheme of thiazolidine formation between salicylaldehyde and the CAL peptide. b) Mass spectrum of the salicylaldehyde-CAL thiazolidine conjugate. c) Stacked ^{1}H-NMR of salicylaldehyde (bottom) and its CAL thiazolidine conjugate.

XVI. Synthesis and characterization of peptides

All peptides were synthesized by using Fmoc-chemistry with the Rink Amide MBHA resin or Wang resin as the solid support. The syntheses were carried out on 0.05 mmole scale. Five equivalents of the commercially available amino acids and HBTU were used for the coupling reaction. The peptides were cleaved off the resin and globally deprotected with reagent K (82.5% TFA : 5% phenol : 5% H_2O : 5% thioanisole : 2.5% EDT). Crude peptides were obtained through ether precipitation and then purified by RP-HPLC (Waters Prep LC, Jupiter C_{18} Column). The peptide sequences and the corresponding mass-spec data are listed in Table S3. ^{1}H-NMR was recorded to confirm the purity of the CAL peptide, and the purity of Cys-VHP was confirmed by LC-MS analysis (Figure S14). The concentration of the CAL peptide was determined by weight, and the concentration of Cys-VHP and helical peptide were determined by UV-vis absorbance at 280 nm.

Table S3. Peptide sequences and observed mass

Peptide	Sequence	Mass (m/z)
CAL	H-CAL-NH_2	Calc. $[M+H]^+$ 305.1642<br>Observed 305.1587
Cys-VHP	H-CGGLSDEDFKAVFGMTRSAFANLWKQQHLKKEKGLF-OH	Calc. $[M+3H]^{3+}$ 1363.9173<br>Observed 1363.6938
H1	H-WAEAAAKCAAAKEAAAKA-NH_2	Calc. $[M]^+$ 1731.0090<br>Observed 1730.8924

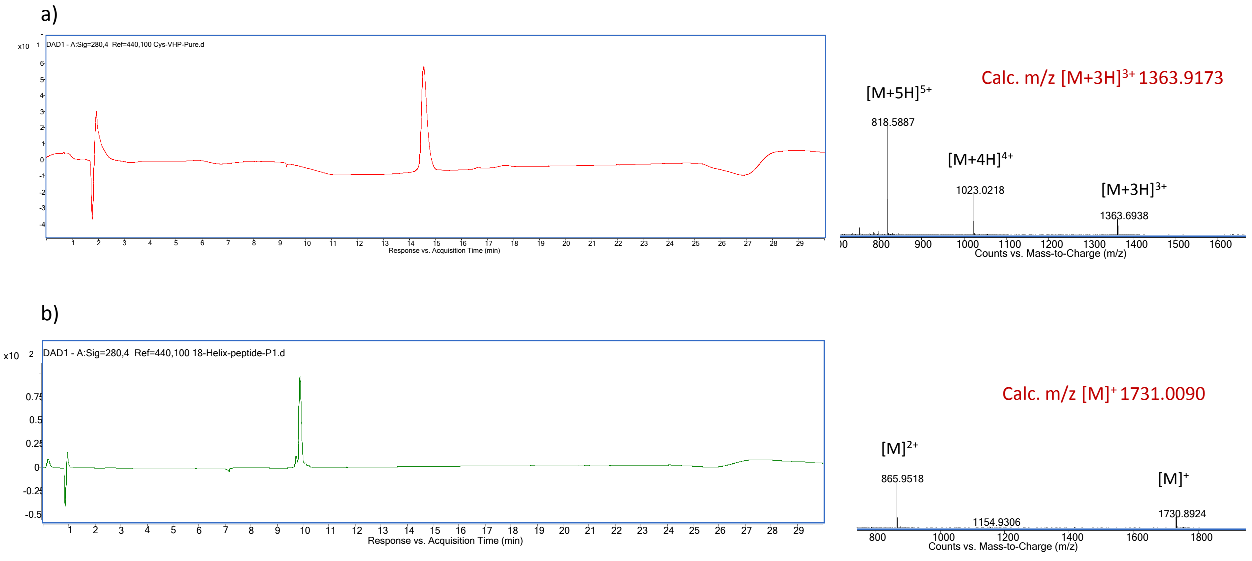

Figure S17. LC-MS characterization of a) Cys-VHP and b) the helical peptide H1. The LC-MS experiments were run using **Method II** (page 2) and the UV-vis detector was set at 280 nm.

XVII. Synthesis of NBD labelled 2-FPBA (2-FPBA-NBD)

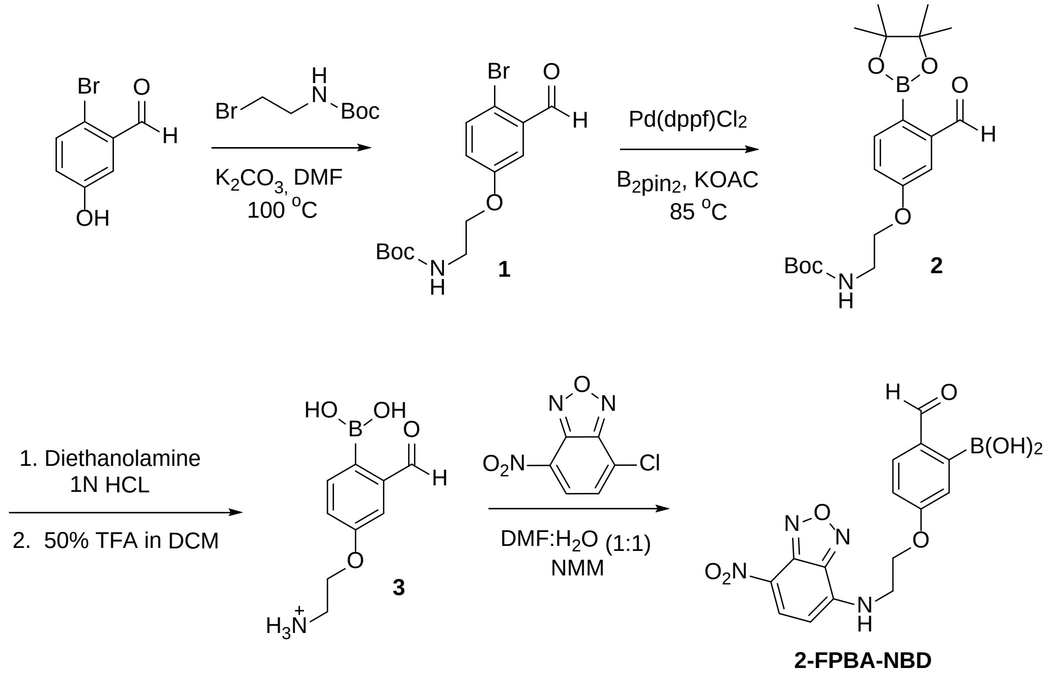

Figure S18. Synthetic route for the NBD labelled 2-FPBA derivative.

<u>*Synthesis of 1*</u>

2-Bromo-5-hydroxybenzaldehyde (1 g, 4.98 mmol) and Boc-NH-$(CH_2)_2$-Br (5.47 mmol, 1.23g) were dissolved together in DMF. Potassium carbonate (14.93 mmol, 2.06g) was added to the reaction mixture, which was then stirred for 17 hours at 100°C. The reaction was monitored by TLC and LC-MS, and at 17 hours, there was only 30% conversion to product. The reaction mixture was then diluted in 80 mL ethyl acetate and washed with water (1 × 80 mL). The aqueous layer was extracted two additional times with ethyl acetate (2 × 80 mL). The combined organic layers were then washed with brine (100 mL) and dried over anhydrous Na_2SO_4. The organic layer was evaporated under reduced pressure and the crude material was then was purified through silica gel using hexane:ethyl acetate (10:1) to yield a yellow oil (502 mg, 29%).

^{1}H NMR ($CDCl_3$) δ: 1.38 – 1.51 (s, 9H), 3.43 – 3.64 (m, 2H), 3.99 – 4.13 (t, *J* = 5.0 Hz, 2H), 4.84 – 5.05 (s, 1H), 6.91 – 7.05 (m, 1H), 7.32 – 7.43 (s, 1H), 7.46 – 7.55 (d, *J* = 8.8 Hz, 1H), 10.19 – 10.33 (s, 1H).

^{13}C NMR ($CDCl_3$) δ: 191.53, 158.21, 155.78, 134.62, 133.98, 122.97, 118.12, 113.73, 79.69, 67.71, 39.87, 28.35.

MS-ESI$^+$: *m/z* calculated for $C_{14}H_{18}BrNO_4$ $[M\text{-}Boc+H]^+$ 243.9973, observed 243.9891.

Synthesis of 2[2]

1 (140 mg, 0.407 mmol) was dissolved in anhydrous dioxane (2.4 mL). B_2pin_2 (260 mg, 1.02 mmol), Pd(dppf) (16.6 mg, 0.020 mmol), and potassium acetate (124 mg, 1.22 mmol) were added into the solution. The reaction vessel was flushed with argon before the reaction mixture was allowed to stir at 85 ^oC for 2 hours. Completion of the reaction was monitored by LC-MS, which revealed that about 40% of the starting material converted to the protodeboronated product. Afterwards, water (50 mL) was added to the reaction and the product was then extracted with ethyl acetate (3 × 50 mL). The combined organic layers were then washed with brine (50 mL) and dried over anhydrous Na_2SO_4. The organic layer was concentrated under vacuum and purified on silica gel using hexane: ethyl acetate (10:1) as eluent to yield a clear gummy product (68 mg, 43%).

^{1}H NMR ($CDCl_3$) δ: 1.32 – 1.40 (s, 12H), 1.39 – 1.47 (s, 9H), 3.49 – 3.58 (q, *J* = 5.4 Hz, 2H), 4.05 – 4.10 (t, *J* = 5.2 Hz, 2H), 4.94 – 5.06 (s, 1H), 7.07 – 7.12 (dd, *J* = 8.3, 2.7 Hz, 1H), 7.45 – 7.46 (d, *J* = 2.6 Hz, 1H), 7.82 – 7.88 (d, *J* = 8.3 Hz, 1H), 10.62 – 10.66 (s, 1H).

^{13}C NMR ($CDCl_3$) δ: 194.72, 160.86, 155.81, 143.53, 138.09, 120.16, 110.97, 84.17, 79.61, 67.30, 39.93, 28.61, 24.76.

MS-ESI$^+$: *m/z* calculated for $C_{20}H_{30}BNO_6$ $[M\text{-}(^tBu+pinacol+H_2O)+2H]^+$ 236. 0730, observed 236.0692.

Synthesis of 3[2]

2 (35 mg, 0.089 mmol) was dissolved in 300 μL THF. Diethanolamine was then added to the reaction mixture (94 mg, 0.890 mmol), which was stirred at room temperature for 14 hours. The reaction mixture was then evaporated under reduced pressure and acidified with 1 mL 1N HCl. The mixture was then stirred for 30 minutes before being extracted with ethyl acetate (3 × 5 mL). The combined organic layers were then washed with brine (1 × 5 mL) and dried over anhydrous Na_2SO_4. The organic layer was evaporated under reduced pressure after which it was dissolved in 5 mL DCM:trifluoroacetic Acid (50:50) and stirred for 30 minutes. Finally, the mixture was evaporated under reduced pressure, dissolved in 1 mL water, and lyophilized. The lyophilized material was then purified via reverse phase HPLC using acetonitrile/water (1% TFA) mixture as eluent to get a white powder after lyophilization. The compound identity was confirmed by LC-MS and it was directly used for NBD labelling without further characterization.

MS-ESI$^+$: *m/z* calculated for $C_9H_{12}BNO_4$ $[M\text{-}H_2O+H]^+$ 192.0859, observed 192.0839.

Synthesis of 2-FPBA-NBD[3]

3 (0.5 mg, 0.0024 mmol) and NBD-Cl (0.6 mg, 0.003 mmol) were dissolved together in 3:2 water:DMF (200 µL). Then 5 µL of 1.5 M NMM in DMF was added into the reaction mixture. The mixture was stirred for 5 hours at room temperature. The reaction turned yellow to brown over time, which is the indication of the progress of reaction. The product was then purified via reverse phase HPLC (Waters Prep LC, Jupiter C_{18} Column) using acetonitrile/water (1% TFA) mixture as eluent and confirmed with LC-MS for integrity and purity using Method II as shown in General Methods. The lyophilized product was further dissolved in phosphate buffer (pH 7) and the stock concentration was determined by using a UV-vis spectrometer with ε (493 nm) = 24,000 $M^{-1}cm^{-1}$.

MS-ESI$^+$: *m/z* calculated for $C_{15}H_{13}BN_4O_7$ $[M-H_2O+H]^+$ 355.0850, observed 355.0845.

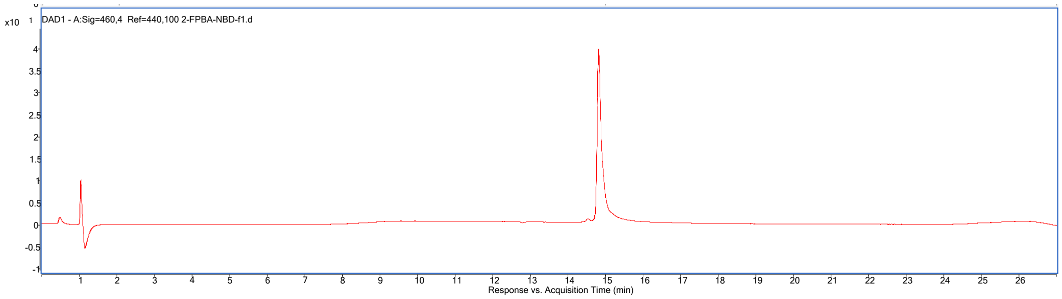

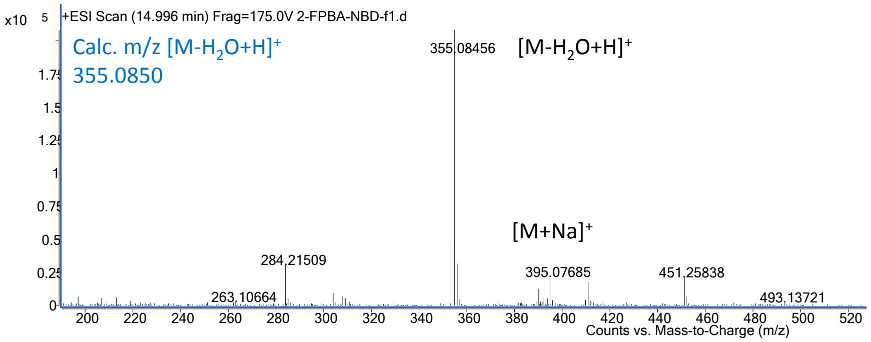

Figure S19. LC trace (top) and mass spectrum (bottom) of 2-FPBA-NBD. The LC trace was recorded at 460 nm with **Method II** described in the General Methods section.

XVIII. Reference

1. L. Wang, C. Dai, S. K. Burroughs, S. L. Wang and B. Wang. *Chem. –Euro, J.*, 2013, **19**, 7587-7594.
2. A. Bandyopadhyay, K. MaCarthy, M. Kelly and J. Gao, *Nat. Commun.*, 2015, **6**:6561.
3. Y. K. Reshetnyak, O. A. Andreev, U. Lehnert and D. M. Engelman. *Prot. Natl. Acad. Sci., USA*, 2006, **103**, 6460-6465.

XIX. NMR spectra

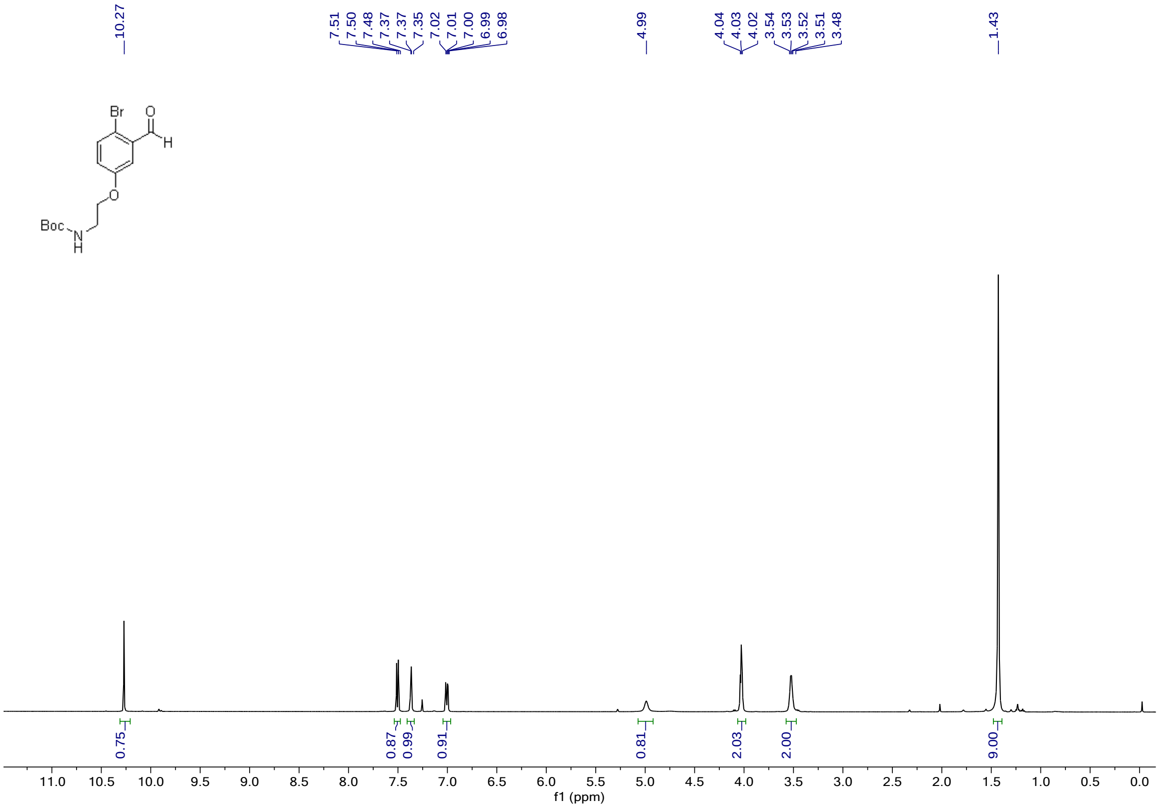

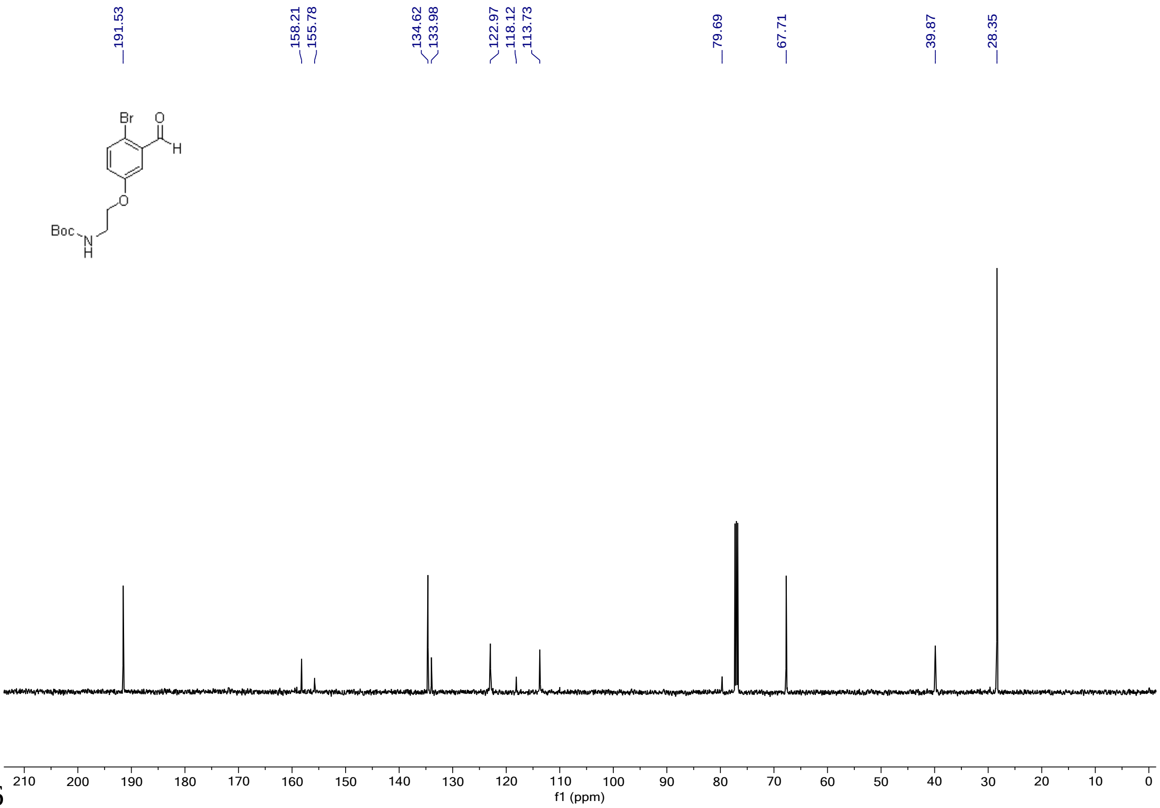

6

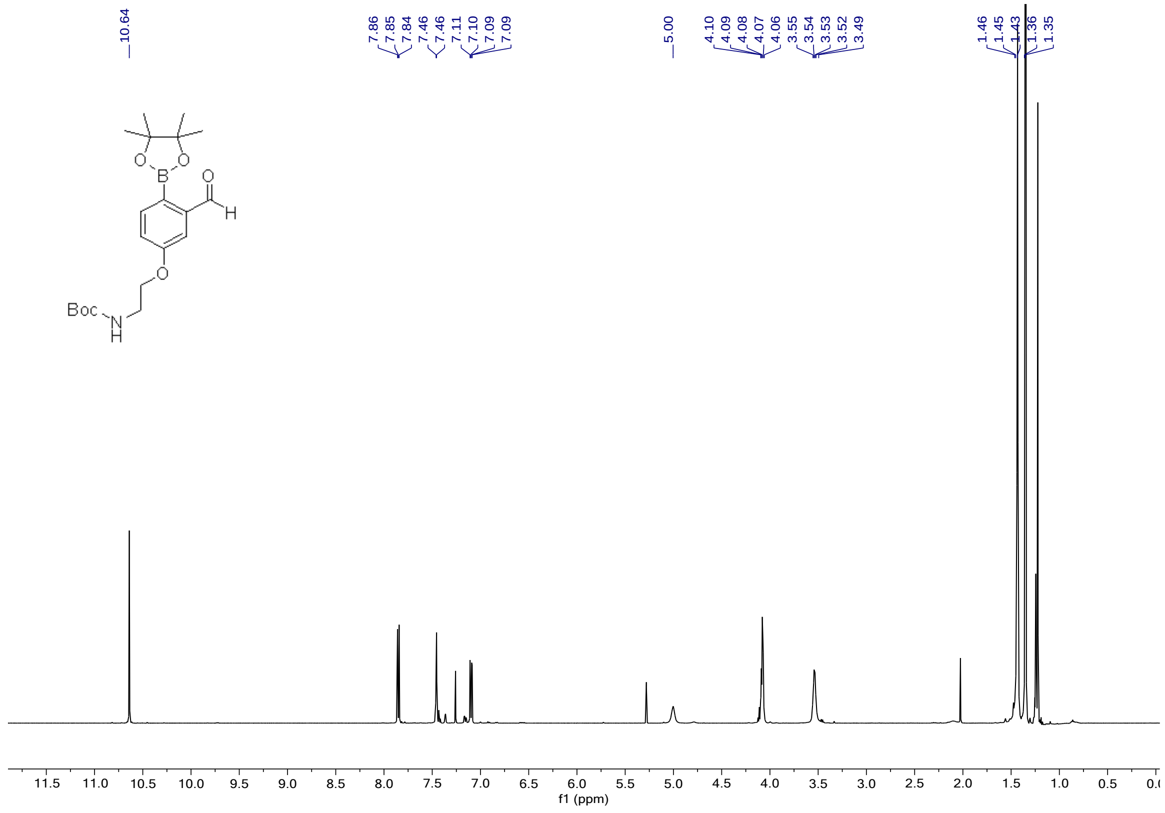
10.64
7.86
7.85
7.84
7.46
7.46
7.11
7.10
7.09
7.09
5.00
4.10
4.09
4.08
4.07
4.06
3.55
3.54
3.53
3.52
3.49
1.46
1.45
1.43
1.36
1.35
O
B
O
O
H
O
Boc
N
H
11.5 11.0 10.5 10.0 9.5 9.0 8.5 8.0 7.5 7.0 6.5 6.0 5.5 5.0 4.5 4.0 3.5 3.0 2.5 2.0 1.5 1.0 0.5
f1 (ppm)

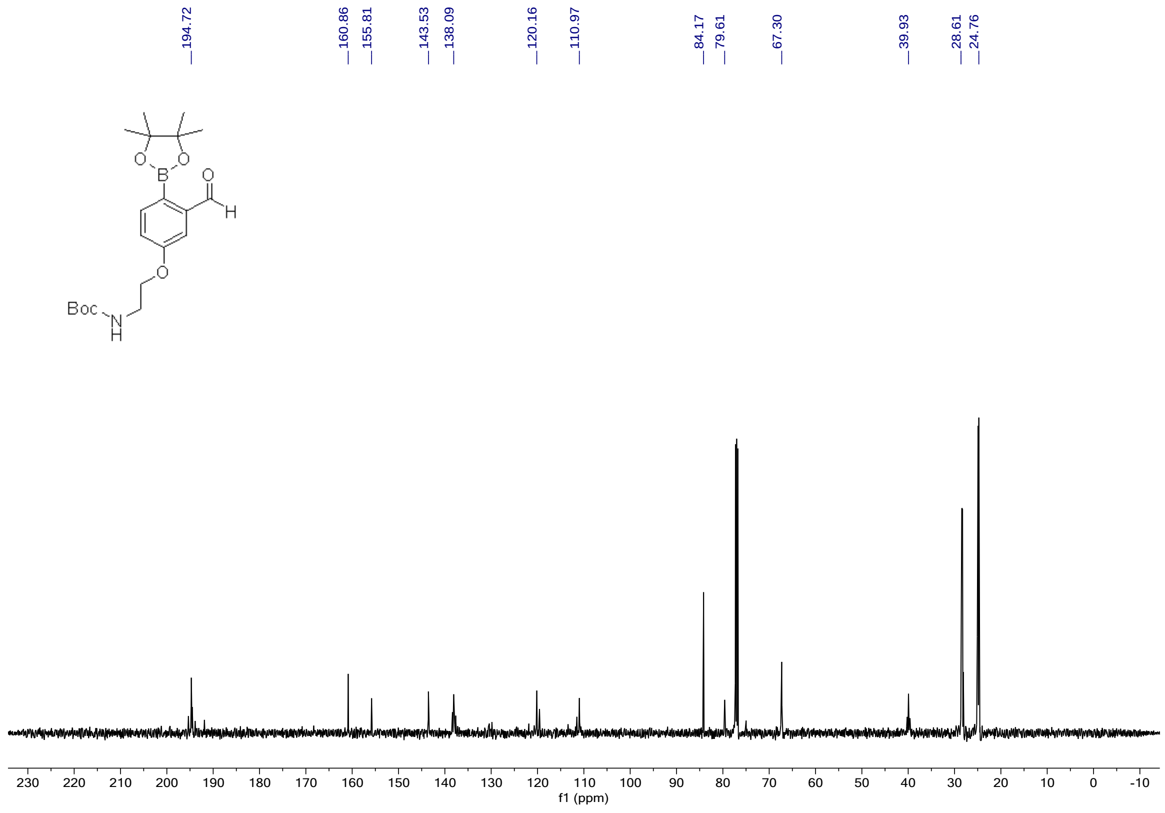
194.72
160.86
155.81
143.53
138.09
120.16
110.97
84.17
79.61
67.30
39.93
28.61
24.76
O
B
O
O
H
O
Boc
N
H
230 220 210 200 190 180 170 160 150 140 130 120 110 100 90 80 70 60 50 40 30 20 10 0 -10
f1 (ppm)
